# Transient social–ecological dynamics reveal signals of decoupling in a highly disturbed Anthropocene landscape

**DOI:** 10.1073/pnas.2321303121

**Published:** 2024-04-19

**Authors:** Qi Lin, Ke Zhang, Charline Giguet-Covex, Fabien Arnaud, Suzanne McGowan, Ludovic Gielly, Eric Capo, Shixin Huang, Gentile Francesco Ficetola, Ji Shen, John A. Dearing, Michael E. Meadows

**Affiliations:** ^a^Key Laboratory of Lake and Watershed Science for Water Security, Nanjing Institute of Geography and Limnology, Chinese Academy of Sciences, Nanjing 210008, People’s Republic of China; ^b^Laboratoire Environnements, Dyamiques et Teritoires de la Montagne, Université Savoie Mont Blanc, CNRS, Chambéry 73000, France; ^c^Department of Aquatic Ecology, Netherlands Institute of Ecology, Wageningen 6708PB, Netherlands; ^d^Laboratoire d’Écologie Alpine, CNRS, Université Grenoble Alpes, Grenoble F-38000, France; ^e^Department of Ecology and Environmental Sciences, Umeå University, Umeå SE-90187, Sweden; ^f^Department of Environmental Science and Policy, University of Milan, Milan 20133, Italy; ^g^School of Geography and Ocean Science, Nanjing University, Nanjing 210023, People’s Republic of China; ^h^School of Geography and Environmental Science, University of Southampton, Southampton SO17 1BJ, United Kingdom; ^i^Department of Environmental & Geographical Science, University of Cape Town, Rondebosch 7701, South Africa

**Keywords:** social–ecological system, critical transition, resilience, freshwater ecosystem, Anthropocene

## Abstract

Understanding the transient dynamics of interlinked social–ecological systems (SES) is imperative for assessing sustainability in the Anthropocene. However, how to identify critical transitions in real-world SES remains a formidable challenge. In this study, we present an evolutionary framework to characterize these dynamics over an extended historical timeline. Our approach leverages multidecadal rates of change in socioeconomic data, paleoenvironmental, and cutting-edge sedimentary ancient DNA records from China’s Yangtze River Delta, one of the most densely populated and intensively modified landscapes on Earth. Our analysis reveals two significant social–ecological transitions characterized by contrasting interactions and feedback spanning several centuries. Initially, the regional SES exhibited a loosely connected and ecologically sustainable regime. Nevertheless, starting in the 1950s, an increasingly interconnected regime emerged, ultimately resulting in the crossing of tipping points and an unprecedented acceleration in soil erosion, water eutrophication, and ecosystem degradation. Remarkably, the second transition occurring around the 2000s, featured a notable decoupling of socioeconomic development from ecoenvironmental degradation. This decoupling phenomenon signifies a more desirable reconfiguration of the regional SES, furnishing essential insights not only for the Yangtze River Basin but also for regions worldwide grappling with similar sustainability challenges. Our extensive multidecadal empirical investigation underscores the value of coevolutionary approaches in understanding and addressing social–ecological system dynamics.

## Significance

In the Anthropocene, critical social–ecological transitions hold profound significance for both environmental stability and human well-being. Our study advances a dynamic-based approach to assess the adaptability or transformative potential of social–ecological systems (SES) through time, by integrating a social–ecological coupling index based on rate of change. Drawing on empirical evidence from sedimentary ancient DNA, biophysical, and socioeconomic records, we explore the evolutionary dynamics of interconnected SES in China’s Lake Taihu watershed over the last century. Notably, we present compelling evidence of unprecedented decoupling signals between socioeconomic growth and ecoenvironmental degradation, particularly in the last two decades. These decoupling trends, crucial for sustainability assessments, may remain cryptic if relying solely on the magnitude of change derived from short-term change metrics.

Achieving the United Nations Sustainable Development Goals by 2030 (1) requires a comprehensive understanding of the deeply interconnected nature of complex social–ecological systems (SES) (2). Escalating environmental changes interact with large social and economic unrest, triggering feedback loops, abrupt changes, cascading failures, and heightened exposure to systemic risks (3, 4). Failure to recognize such couplings and nonlinear effects has led to pressing environmental issues and the inefficacy of proposed solutions. Embracing an SES perspective is imperative to reveal crucial elements that might otherwise go unnoticed, such as coevolving dynamics, system thresholds, and criteria essential for transformative change. This approach serves as the key to identifying and pursuing pathways toward sustainable development in a dynamic and uncertain Anthropocene era (5–7).

Characterizing the nonasymptotic or transient dynamics of SES has substantial implications for transforming society into a more desirable future. Typically, socioeconomic development is coupled to a heavily altered and degraded ecoenvironmental state (8–10), often resulting in undesirable SES configurations associated with phenomena such as the “poverty trap” (11). The United Nations’ 2030 sustainability agenda calls for “bold and transformative steps which are urgently needed to shift the world onto a sustainable and resilient path” (1), to escape the social–ecological traps and prevent catastrophic collapse (12). Achieving this transformation requires a fundamental decoupling of economic growth from ecoenvironmental degradation (13).

**Fig. 1.**
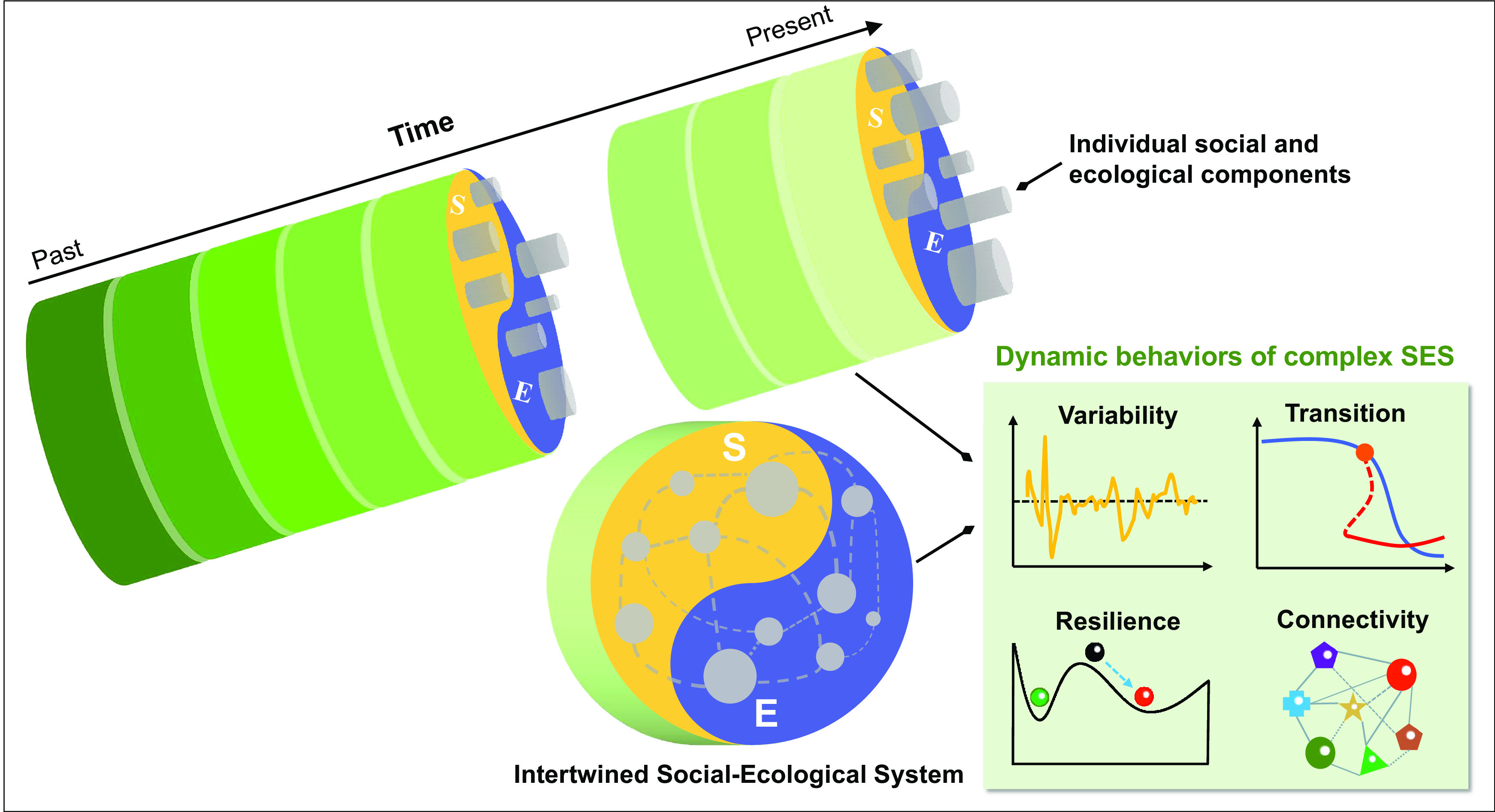
Schematic diagram of dynamic changes of intertwined SES from a historical evolutionary perspective. The dynamic relationship between social and ecological systems is depicted, emphasizing their interdependence and coevolutionary trajectory. By integrating long-term social and biophysical empirical data within an evolutionary framework, this diagram serves as the basis for exploring critical inquiries into the functionality of regional SES. This evolutionary perspective offers valuable insights into the transient behaviors of complex systems, contributing to sustainability discourse in the Anthropocene.

However, transient system trajectories to an alternative stable state may vary substantially, posing considerable challenges in discerning between more or less favorable transformation pathways (14). Present knowledge of transient dynamics primarily focuses on either ecological or social systems through modeling or conceptual analysis (15, 16), with limited progress in identifying and conceptualizing SES as intricate networks of social and ecological interdependence (6). This limitation stems in part from the absence of integrated generic variables that can capture the transient dynamics of interlinked SES. Moreover, it is increasingly recognized that many SES transitions are embedded within longer-term dynamics, spanning from multidecadal to centennial scales (17, 18). In the absence of sufficient empirical evidence on these temporal dynamics, critical issues like the timing of abrupt transitions, the underlying characteristics of transient behaviors, and the identification of potential driving factors (19), remain inadequately addressed (Fig. 1). A comprehensive understanding of the mechanisms through which pathways of persistence, adaptation or transformation emerge and evolve is crucially required (20). Furthermore, there is a growing call to incorporate a rate-focused framework to address unprecedented challenges in the Anthropocene (21), alongside traditional state-focused research that aims to manage desired states from a static perspective. Short-term static state variable-based assessments fail adequately to capture longer-term patterns and processes, and easily overlook nonlinear behaviors and hysteresis effects of slow trends driven by systemic feedback interactions (22). While there is increasing focus on rate of change (RoC) in studying ecosystem, climate, social processes (e.g., refs. 23–25), the application of the RoC in integrated SES research remains limited.

**Fig. 2.**
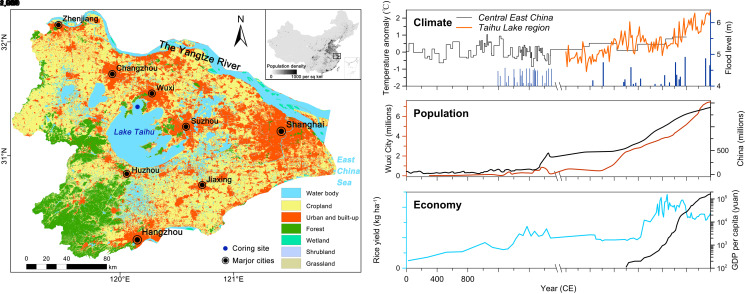
Context for sustainability challenges of Lake Taihu watershed in China’s Yangtze River Delta. The *Left* panel displays maps of human population density in China (source: https://www.worldpop.org/) and land-use cover of Lake Taihu watershed (source: https://env1.arcgis.com/arcgis/rest/services/Sentinel2_10m_LandCover/ImageServer) for the year 2020, illustrating the densely populated and strongly human-shaped nature of the region. The *Right* panel presents multidecadal to multicentennial changes of temperature anomaly, flood level, human population, rice yield, and GDP per capita. Detailed information about the data compilation can be seen in *Materials and Methods*.

In this study, we demonstrate how to track transient dynamics in the interconnected social–ecological system over the past centuries in China’s Yangtze River Delta, one of the world’s most densely populated and intensively modified landscapes (Fig. 2). As a symbol of China’s booming economy, this region has undergone unprecedented social and ecological upheaval, and now confronts pressing challenges in transitioning toward a sustainable development path (26–29). How to develop a holistic understanding of the system’s dynamic nature so that adaptive management strategies can be employed to ensure sustainability remains a critical question facing decision-makers in the context of such turbulent and shifting SES. Hence, our study employs an empirical, evolutionary approach to reveal the nonlinear dynamics of a rapidly changing iconic SES in the watershed of Lake Taihu, the third-largest freshwater lake in China, where the lake restoration dilemma is still rooted in the conflict of economic growth and pollution governance (30).

By integrating the application of sedimentary ancient DNA (*sed*aDNA) metabarcoding and multiproxy paleoenvironmental analyses with social and economic empirical records, we systematically evaluate the interactive impacts of progressive agricultural transformations, increasing urbanization, and public policy initiatives on multidecadal trajectories of air quality, soil stability, terrestrial vegetation succession, and aquatic ecosystem health. This coevolutionary approach allows us to address their dynamic relationships and feedback mechanisms comprehensively. More importantly, we develop an integrative index based on the RoC (31) and coupling model (10, 32) that characterizes transient dynamics of the regional SES (*Materials and Methods*). Our analysis facilitates a reassessment of the trajectories and dynamic behaviors of the interconnected SES, providing an opportunity to evaluate the potential for decoupling economic growth from ecoenvironmental degradation. Such insights are crucial for shaping a more context-sensitive sustainability agenda that fosters favorable transformations at local to global scales.

## Results

**Fig. 3.**
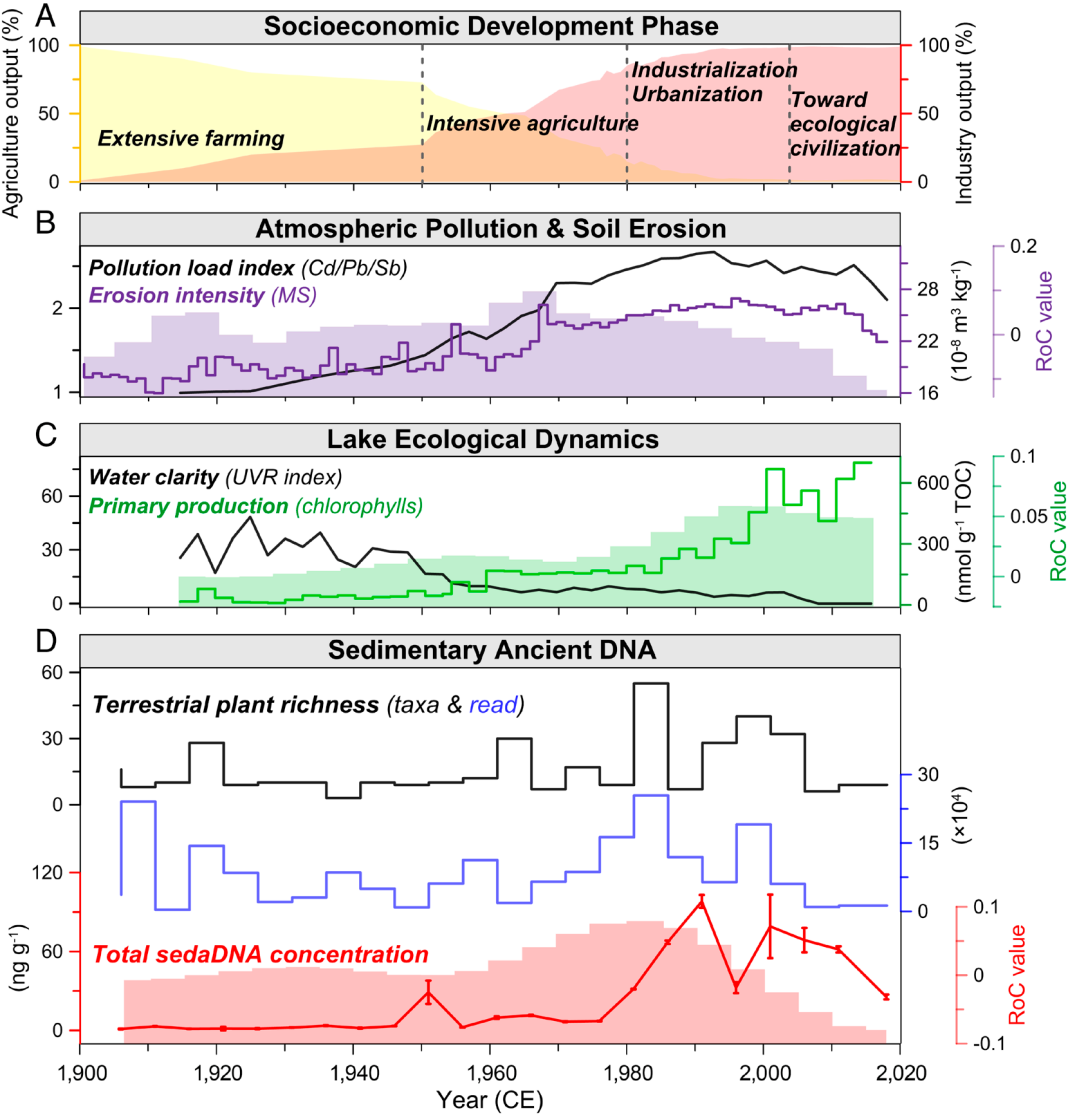
Century-scale socioeconomic and ecoenvironmental trajectories of Lake Taihu watershed. (*A*) Socioeconomic development, represented by gross agricultural output (yellow) and industrial output (red) as a percentage of GDP, is divided into four phases (dashed lines). (*B*–*D*) Trajectories and RoC of multiple biophysical variables are shown. These include mass magnetic susceptibility (MS), pollution load index of atmospheric trace metal deposition (Cd, Pb, Sb), chlorophylls (sum of chlorophyll-a and pheophytin-a), ultraviolet ray (UVR) index inferred from algal pigments, total sedimentary DNA concentration, and the numbers of total taxa and DNA reads of terrestrial plants. In figures (*B*–*D*), color-coded bar graphs represent the RoC results for erosion (purple), primary production (green), and *sed*aDNA (red). The RoCs provide valuable insights beyond the state indicators. For example, in figure (*C*), while state variables indicate high levels of algal production in recent decades, the RoC analysis reveals a gradual decrease, signaling an early indication of potential water degradation deceleration.

**Fig. 4.**
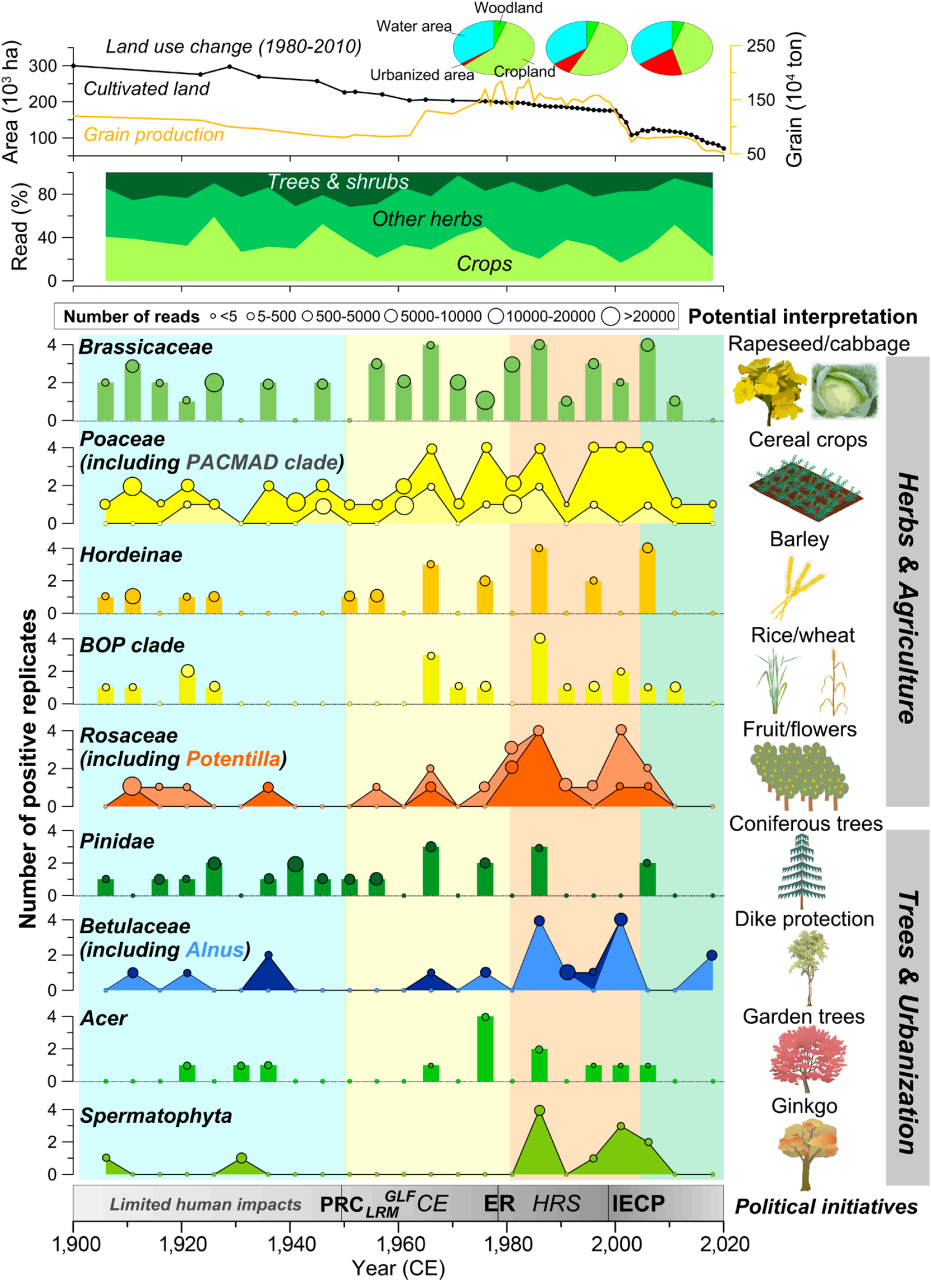
Dynamics of terrestrial plant *sed*aDNA over time in relation to agriculture and urbanization. The community composition of terrestrial plants (tree & shrub, crop, and other herb) is inferred from the percentage of the number of sequence reads for different groups. The taxa presented here, using the numbers of positive PCR replicates (values on the *y*-axis) and total sequence reads (size of circles), are selected to show the relation between plants and local human activities (agriculture practices, urbanization, and related public policies). Four specific phases of changes in plant *sed*aDNA were defined (vertical shaded areas) and discussed in the text, which corresponds to the changes in sedimentological and geochemical characteristics (*SI Appendix*, Fig. S3), and *sed*aDNA records (Fig. 3 and *SI Appendix*, Fig. S8) for potential interpretation. At the *Top*, cultivated land area, grain production, and land-use data in 1980, 2000, and 2010 are shown. At the *Bottom*, public political initiatives (horizontal bar) include the foundation of P.R. China (PRC), the Land Reform Movement (LRM), the Great Leap Forward (GLF), the collectivized economy (CE), the economic reform (ER), the Household Responsibility System (HRS), and the implementation of ecological conservation programs (IECP) (26, 29). Deeper gray of the horizontal bar generally indicates stronger human disturbance with negative ecological effects.

**Fig. 5.**
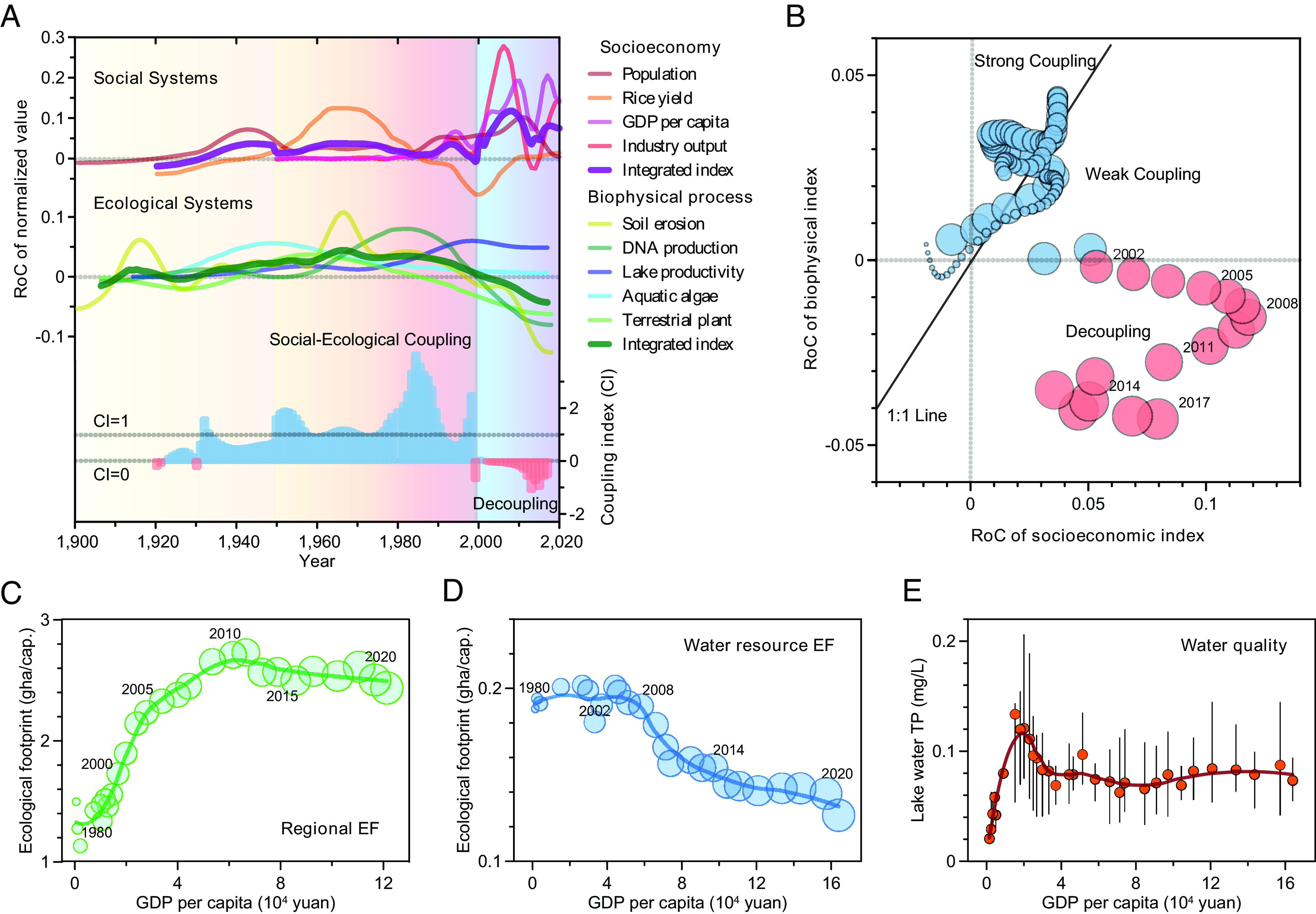
Integrated coupling-decoupling dynamics in the interlinked SES. (*A*) RoCs for different socioeconomic and biophysical time series (for details, refer to *SI Appendix*, Figs. S11–S13 and Tables S1 and S2). The thick purple and dark green lines represent the integrated RoC index of the social and ecological systems, respectively. The coupling index (CI) in the *Bottom* panel shows variations of coupling degree between socioeconomic development and ecoenvironmental degradation. When 0 < CI < 1, the rate of ecoenvironmental degradation lags behind that of socioeconomic development within a sustainable regime; when CI ≥ 1, the rate of ecoenvironmental degradation keeps pace with or is higher than socioeconomic development within an unsustainable regime; when CI = 1, it is the balance point between absolute coupling and relative decoupling; when CI = 0, it means the socioeconomy is developing, while ecoenvironmental status remains stable; when the ecological condition improves while the society keeps developing in a desirable and more sustainable path, then CI < 0. (*B*) Relationships between integrated RoCs for socioeconomy and biophysical process within the SES over the last 100 y. The transition process from coupling to decoupling (red) interactions is illustrated by the 1:1 line. (*C*) Relationships between regional consumption-based ecological footprint (EF) per capita and GDP per capita through the period of 1980 to 2020 with LOESS fit. (*D*) Relationships between water resource EF per capita and GDP per capita in Lake Taihu watershed over time with LOESS fit. (*E*) Relationships between monitored lake water total phosphorus (TP) concentration and GDP per capita in the watershed over time. In figures *B*–*D*, the size of each data point represents the corresponding date (year), which is labeled around the data point. These consumption-based EF in terms of human land-use and water resources generally showed increasing then decreasing trends (particularly from the early 2010s), along with the continued economic growth over time.

Comprehensive analyses of multisource archives disclose century-scale natural and anthropogenic modifications of land surface processes, hydrological and ecological dynamics in the Lake Taihu watershed. Paleoenvironmental proxies for anthropogenic pollution, soil erosion, water eutrophication, algal and plant communities highlight prominent changes through time (Figs. 3 and 4 and *SI Appendix, Text S3–S5*). The transient system dynamics, revealed by integrating the historical variability (i.e., RoC) of societal and economic transformations and biophysical processes in a coupling model, indicate significant social–ecological transitions (Fig. 5 *A* and *B* and *SI Appendix*, Fig. S11). Our results suggest that the highly intertwined system has undergone four evolutionary phases (Figs. 3 and 4), characterized by particular combinations of nonlinear interactions and feedback (*SI Appendix*, Fig. S13).

Prior to the 1950s, relatively low values of magnetic susceptibility (MS) suggest minimal catchment soil erosion in the context of a relatively primitive, traditional agriculture economy (33) and lower flood levels (Fig. 2). In this phase, total and plant *sed*aDNA, especially that associated with most crop types, was maintained at a low abundance (Fig. 4). Sediment pigment indicators suggest that the lake ecosystem sustained a clear-water, less productive state. Correspondingly, the coupling index (CI) derived from integrative modeling is generally <1 for the regional SES (Fig. 5*A*), indicating that socioeconomic development during this period did not have significant deleterious consequences for ecosystem integrity.

Following the foundation of the Peoples’ Republic of China, increasing MS, sand fraction, and peak values of zirconium-to-rubidium (Zr/Rb) ratios point to enhanced soil erosion characterized by greater transport of coarse particles to the lake during the 1950s to 1980s (Fig. 3 and *SI Appendix*, Figs. S3, S6, and S7). This trend is associated both with agricultural development, inferred from *sed*aDNA signals, and hydrodynamic fluctuations, suggesting that these effects combined to augment erosion dynamics. Increased occurrences of DNA related to crop cultivation, including rapeseed and/or cabbage (Brassicaceae), and cereal taxa (maize, rice, wheat, barley, etc.) (e.g., Poaceae, *PACMAD* clade, *BOP* clade, Hordeinae), are explicitly recorded (Fig. 4). Meanwhile, anthropogenic trace metal contamination increased (*SI Appendix*, Fig. S7) as did lake primary productivity, which may in part be the result of erosion of enriched and more polluted soils (34). Statistical breakpoint analyses using sequential *t* test algorithm and cumulative sum of differences for major biophysical parameters indicative of air quality, soil stability, water quality, and plant communities, reveal significant tipping points and potential regime shifts during the 1950s to 1980s (*SI Appendix*, Fig. S10), suggesting rapid, large, and nonlinear changes in the environmental systems (35). The RoCs of soil erosion and vegetation also reached maximum values during this phase (Fig. 5*A*), which can be interpreted as generic signs of decreased land system stability and loss of resilience (36). The integrated system shows a suddenly elevated CI of approximately 1, reflecting a stable and strong stage of coupling between agricultural development and ecological degradation.

Since China’s economic reform and opening up in 1978 CE, there is evidence of a pronounced decrease in sediment Zr and Zr/Rb ratios that suggests reduced erosion, especially of coarse particles (*SI Appendix*, Figs. S3 and S7). Increased quantities of DNA from Betulaceae (including *Alnus sp.*), *Taxus sp.*, and *Broussonetia papyrifera* (Fig. 4) are indicative of plantations introduced for dike protection and erosion control along the lakeshore and riverside. This is related to the “Shelter-belt Program for Huaihe River and Lake Taihu” that was implemented during 1992 to 2000 CE, which led to increased forest cover by ~4% (37). Meanwhile, intensive agriculture and urbanization continue to be recorded in sediment DNA and geochemistry. In particular, taxa from the Rosaceae family may reflect fruit cultivation. Certain ornamental arboreal taxa, such as *Acer sp.*, Caprifoliaceae (including *Lonicera sp.*), and Spermatophyta (including Ginkgo) (38), exhibit increased abundance in parallel with the expansion of urbanized land and associated greenbelts (Fig. 4 and *SI Appendix*, Fig. S8*D*). Contemporaneously, however, the aquatic ecosystem experienced catastrophic degradation, characterized by excess nutrient enrichment, decreased water clarity, and the development of harmful algal blooms (Fig. 3 and *SI Appendix*, Figs. S3 and S5) (39). The reduced and even negative RoCs of rice yield suggest an overall loss of agroecosystem sustainability, while variability and vulnerability of the lake ecosystem itself actually increased, as indicated by significantly and/or increasingly positive RoCs for primary productivity and algal community turnover (Fig. 5*A*). In this phase, society’s ecological footprints (EFs) and complexity of interactions are augmented due to pressure exerted by accelerating population density. Correspondingly, the system CI is maintained at high levels (CI > 1) and reached the maximum values in the middle 1980s.

In the 21st century, although the regional population and economy have increased notably, a surprising decline in the values of minerogenic proxies associated with erosion (e.g., Ti, Zr, MS) is exhibited, along with more limited plant taxa and DNA reads. Marginal increases in the area of both freshwater and woodland in the catchment are observed (by 0.13% and 0.12%, respectively), while cropland and grain production are markedly reduced (Fig. 4), largely due to the implementation of conservation programs under the policies aimed at achieving an “ecological civilization” in China (40). In contrast, the lake continues to be plagued by phosphorus enrichment and toxic cyanobacterial blooms despite massive environmental management investment (41), suggesting ongoing degraded status and larger recovery hysteresis of the aquatic ecosystem (Fig. 3). There are sharp increases in the RoCs related to the industrial economy, whereas the RoCs of major biophysical variables including lake ecological indicators exhibit decreasing trends to varying degrees. Notably, a transient flickering-like phenomenon (15, 42) and the decrease of CI below 0 are apparent, highlighting the occurrence of a critical transition from regional coupling to decoupling (Fig. 5 *A* and *B*) that may pre-empt the emergence of synchroneity in socioeconomic development and ecological conservation.

## Discussion

Our study examines the dynamic trajectories, current phase, and underlying feedback of the SES using multidecadal RoCs and status indicators for key socioeconomic and biophysical processes. This approach enables us to explore the interdependence and transient dynamics of the system’s components, often overlooked in empirical studies of coupled systems (15, 17, 43). Focusing on RoC uncovers perspectives and insights regarding SES dynamics, complementing the studies that focus only on state variables related to magnitude of change, and is reflected in increased rate-focused research across various disciplines in recent years (21, 24, 25). For example, although algal production and water turbidity in Lake Taihu were still at a high level indicated by state variables, the underlying RoCs exhibit gradually decreasing trends, suggesting early signs of the potential slowdown of water degradation over the last decade (Fig. 3). Based on this evolutionary and networked framework, we have identified two significant social–ecological transitions with contrasting dynamic characteristics throughout the past several centuries. Specifically, multiple lines of empirical evidence reveal early signals of decoupling socioeconomic development from ecoenvironmental degradation since the early 2000s (Fig. 5), a pattern not observed over the past millennium.

China’s Lake Taihu watershed was one of the world’s most productive and stable agroecosystems during preindustrial history, and agriculture practices have coevolved with landscapes over millennia in response to changing environmental contexts and social needs (44, 45). Agricultural yield maintained surpluses and supported the regional population that increased about seven-fold through the Common Era until the mid-20th century (Fig. 2), apparently without affecting the stability of the agriculture landscapes (33). The regional SES maintained a dynamic equilibrium with high resilience, the so-called “green-loop”, which renders it capable of anticipating, buffering, and responding to disturbances (8, 29). However, this balance started to disintegrate after “New China” emerged around the 1950s, leading to a shift of the regional SES from a traditional agroecosystem into an intensively productive one that led to substantial degradation of the environment. Despite the unprecedented scale of agroecosystem transformations, the aggregated index suggests that the highest level of coupling between socioeconomic development and ecoenvironmental degradation occurred somewhat later, following the introduction of economic reform in the 1980s. Since then, society’s footprints and complex interactions grow, often with hysteresis, unexpected outcomes, and surprises (43). Agricultural intensification, rural poverty alleviation, and urbanization have been bound together (27), resulting in multiscale landscape transformations, while nevertheless precipitating substantial impacts on local and, ultimately, regional biogeochemical cycles (e.g., anthropogenic nutrient enrichment). Patterns of coupling evident in the past also invoke significant legacy effects, on modern and possibly future conditions (43). In particular, cultural eutrophication associated with the collapse of lake ecosystem and the frequent occurrence of toxic cyanobacterial blooms reflect a catastrophic ecological shift under the pressures of an increasingly urbanized catchment which, in turn, threaten ecosystem service functions of water security and biodiversity.

Since the early 2000s, we have observed a second transition characterized by strong decoupling signals, contrasting with the previous transition that exhibited enhanced coupling. This suggests fundamental changes in the underlying relationships within the SES. Between 2000 and 2020, regional GDP (gross domestic product) increased approximately eight-fold, and the rate of catchment urbanization in this region rose from 50 to ~82%. However, long-term dynamic indicators (RoC) for most ecological systems exhibit decelerating trends (Fig. 5*A*) along with a recent reversal of some state variables (Fig. 3), indicating a marked decline in land-use impacts during this period. Moreover, the consumption-based EF in terms of regional land-use and water resources has plateaued and declined over the past several decades (*SI Appendix*, Fig. S14), which showed significant deviation from the ever increase in economy (e.g., per capita income), particularly since the early 2010s (Fig. 5 *C*–*E*). These lines of evidence suggest the regional SES is transitioning to another state with different underlying mechanisms, possibly driven by new interactions and feedback, avoiding falling into the sustainability trap (12). Nevertheless, whether this process produces a virtuous cycle remains to be seen, most likely depending on further implementation of sustainability policies and transformative steps.

As a harbinger of China’s development, the Yangtze River Delta region has witnessed unprecedent social progress and economic prosperity accompanied by critical environmental degradation. Meanwhile, the local and national authorities have implemented wide-ranging environmental laws and policies, launched conservation and ecological engineering projects, and driven institutional innovations toward promoting sustainability (30, 37, 40). While some studies suggest a decoupling trend for environmental performance from socioeconomic changes (29, 32, 46, 47), others argue that there has been no noticeable recovery despite intensive management efforts (e.g., refs. 27, 30, 41, 48). This ongoing disparity in findings leaves the current state of the linked SES open to interpretation. Most of these studies concentrate on individual sectors, like energy consumption, pollutant and carbon emissions, and GDP (32, 45), with a static view over short time periods (less than 20 y) (47). Our findings based on multiple social and ecological indicators, indicating early signals of regional decoupling at a multidecadal scale, provide holistic insights from an evolutionary social–ecological system perspective. Technological advances and increased dependency on telecoupling may also contribute to regional SES transformation (28), although they may carry the risk of human footprints transferal at a wider scale (3, 4). For instance, the Yangtze River Delta region increasingly depends on importing food and natural resources to fuel development while transferring/relocating some heavy industries to less developed western China and other regions (27, 29), which may have undesired consequences, spillover effects or transition effects on other distant SES. The detailed consequences of telecoupling or spillover effects need to be further investigated by comparing multiple SES changes at more broader scale (28). The complex causal effects and trade-offs between food production, water security, soil stability, and climate change should be given full consideration when operationalizing regional safe and just operating spaces for landscape sustainability (49, 50).

In summary, we present an innovative analytical framework for identifying transient dynamics of interconnected SES through time, by integrating multidecadal RoCs for socioeconomic and biophysical processes in a coupling model. Despite some data limitations (*SI Appendix, Text S6*), our incorporation of reconstructed time series of key ecosystem processes and analytical tests for diverse system behaviors provides a cross-validated evidence base for integrating complex system science and resilience theory with adaptive management (51), potentially leading to a “good Anthropocene” (7, 52). This iconic empirical case study yields valuable insights as to how the intertwined SES has coevolved with different emerging development trajectories/pathways through time, some of which may be undesirable, while others transform and reconfigure the system toward a more sustainable future. These findings can assist decision-makers in better conceptualizing the current state and trajectory of their SES, evaluating the outcomes of management decisions, and enabling adaptive management over time. Our study underscores the importance of adopting a longer-term evolutionary perspective in SES research and stewardship.

## Materials and Methods

### Study Area.

Lake Taihu (N30°55′40″–31°32′58″, E119°52′32″–120°36′10″), China’s third-largest freshwater lake (~2,338 km^2^, mean depth ~2 m), is located in a vast (~36,500 km^2^), densely populated (>40 million inhabitants), and rapidly developing watershed in the floodplain of the Yangtze River Delta (Fig. 2 and *SI Appendix, Text S1* and Fig. S1). Since the 1980s, the annual growth rates of regional GDP, population, and urbanization are approximately 15.7%, 3.0%, and 9.2%, respectively (53). Inflowing rivers are mainly distributed in the north and west of the lake, whereas outflows mainly occur on the eastern side. Dominant soil types include yellow-brown soil, red soil, and paddy soil. Currently, cropland, urban and building land, and waterbodies account for 47.9%, 24.3%, and 13.6% of the total watershed area, respectively (*SI Appendix*, Fig. S2).

The northern lake-watershed region is the most intensively shaped by humans, where two large cities (Wuxi and Changzhou), each with over ten million population are highly developed (Fig. 2). The detrimental effects of various anthropogenic interventions including forest clearance, land reclamation, agricultural intensification, urbanization, and pollution discharge have threatened the regional ecological and social systems for several decades (34, 41). In May 2007, for example, a massive toxic cyanobacterial bloom overwhelmed the water treatment plants that supply Wuxi city, depriving millions of residents of potable water for nearly a week, leading to a highly publicized crisis (54). Although more than 100 billion RMB (~US$14 billion) has been invested in pollution control and water quality improvement during recent decades, the severity of nutrient pollution and cyanobacterial blooms has not lessened as expected from the abatement efforts (41).

### Climate and Socioeconomic Data Compilation.

Instrumental climate data in the Lake Taihu region from 1950 CE, including annual average temperature and total precipitation, were obtained from nearby meteorological station #58358 (N31.06°, E120.43°) from China Meteorological Data Sharing Service System (https://data.cma.cn/). Annual temperature data during 1900 to 1950 CE for the region are sourced from the global historical climatology network (https://www.ncei.noaa.gov/products/land-based-station/global-historical-climatology-network-monthly). Temperature changes during 1900 to 2020 CE were compiled as temperature anomalies from the 1900 to 1950 CE average. Additionally, temperature anomalies with respect to the 1851 to 1950 CE climatology over the past two millennia in Central East China, were reconstructed from historical documents and proxy series (55). Extreme flood-level records during the past millennium were compiled from flood stele and hydrology stations in the lake (56). The monthly average concentration of water total phosphorus in Lake Taihu and annual total water consumption in the watershed monitored since 1980 CE were obtained from the Taihu Basin Authority, Ministry of Water Resources of China.

Datasets for the long-term (multidecadal to millennial) social and economic status in the study area, including annual population, GDP, gross output values of agriculture and industry, arable area, grain and aquatic product production, and rice yield, were compiled from official statistical data of Wuxi city (https://tj.wuxi.gov.cn/) and China (https://www.stats.gov.cn/), the Taihu Basin Authority, and historical documents (33, 57). Land use data within a 40 km-radius buffer from the coring site (sedimentary source region) (Fig. 2), were interpreted from Landsat (TM/ETM+) images in 1980, 2000, and 2010 CE with a spatial resolution of 30 m (http://lake.geodata.cn/) (*SI Appendix*, Fig. S2), following previously validated procedures (53).

## Sediment Archives and Paleoproxy Analyses

Two parallel surface sediment cores (~50 cm) were retrieved from the center (N31°28′52″, E120°10′44″) of Meiliang Bay in the most developed north Lake Taihu watershed using a Kajak sampler (Fig. 2). The geochronology (~110 y) of one core (TH1) based on radionuclide dating technique (^226^Ra, ^210^Pb, and ^137^Cs) has already been reported and published (34, 39). Sediment samples of this core were analyzed at 0.5 to 1 cm contiguous intervals for major physicochemical parameters including water content, grain size, mass MS, total organic carbon, total nitrogen and phosphorus, trace metals, and biotic subfossil proxies of plant pollen and algal pigments (see detailed description in *SI Appendix, Text S2* and Figs. S3–S5). The other parallel core (TAI-18-02; N° IGSN TOAE0000000329; https://cybercarotheque.fr/index.php?sample=13884) was used for sediment geochemical and DNA analyses, and its chronosequence was determined through core matching (based on lithology and geochemistry) with the sediment record from TH1. This core was first split, photographed and logged in the lab, whereafter X-ray fluorescence (XRF) analysis was performed on the sediment surface at 2-mm intervals using a nondestructive Avaatech core-scanner. Principal component analysis was applied to transform the multivariate XRF data into three groups that highlight element correlations and geochemical end-members (*SI Appendix*, Figs. S6 and S7)

From the split sediment core, 23 semicircular slices of 1-cm thickness were sampled with a focus on *sed*aDNA analysis during the past 100 y, following strict laboratory precautions (58). Extraction and amplification of extracellular DNA, which is usually adsorbed within fine sediment particles (mainly clay) enabling protection against nuclease degradation (59), were performed in two separate rooms that were specifically dedicated to ancient DNA analyses. The DNA was extracted from wet subsamples using saturated phosphate buffer (0.12 M Na_2_HPO_4_; pH ≈ 8) and the NucleoSpin^®^ Soil Kit (Macherey-Nagel, Germany) following a modified protocol from ref. 60 and deposited on protocol.io (61). One sampling control and four extraction controls were performed. The extracted *sed*aDNA was quantified using the QuantiFluor^®^ dsDNA System (Promega, USA) and expressed as nanogram DNA per gram dry sediment (ng g^–1^). Vascular plant DNA was amplified with the universal primer pairs *"g-h"* that targeted the P6 loop region of the chloroplast *trn*L (UAA) intron (62). All samples and controls underwent four PCR replicates to improve the reliability of the detection/nondetection pattern. Eight PCR negative controls containing PCR mix but no DNA template were carried out, and randomly distributed on PCR plates. Sequencing was performed by 2*100-bp pair-end sequencing on an Illumina HiSeq 2500 platform.

DNA sequences were filtered using the OBITOOLS software (63) and were assigned to relevant taxa using the ecoTag program based on a suitable database from Genbank, all following the strict protocols (58). We retained DNA reads only with a match of >95% similarity with the sequence in the reference database and detected in at least two consecutive samples presenting a positive PCR. A PCR was considered as positive when a minimum of five read numbers were found for the plant taxa. We focused on terrestrial plants to document watershed surface processes for this study. Vertical migration (leaching) of *sed*aDNA was previously reported as minimal in lake sediments after compaction (64), suggesting that it allows accurate temporal reconstruction.

Following high-throughput sequencing and standard bioinformatic filtering procedures, we obtained 1,920,292 usable merged DNA reads from 92 PCR replicates of sediment samples, corresponding to 98 different sequences of terrestrial plants (including herb, shrub, and tree taxa). After summing sequences assigned to the same taxa and validating the sequences with the “Flora of China” (http://www.iplant.cn/frps) as listed in Jiangsu and Zhejiang provinces around the lake (*SI Appendix*, Figs. S1 and S2) and sediment pollen records in and close to the lake (*SI Appendix*, Fig. S4), 57 adequately identified taxa among 23 samples (from 3 to 55 taxa per sample) (*SI Appendix*, Fig. S8) were retained and used to investigate watershed vegetation dynamics over the past 100 y.

## Numerical and Model analyses

### Community structure change and tipping point analysis.

Multivariate analyses were conducted using Excel and R software with the “vegan” (65) and “mgcv” (66) packages. Nonmetric multidimensional scaling (NMDS) analysis based on Bray–Curtis pairwise distance was used to visualize dissimilarities in plant *sed*aDNA data [log(N+1) transformation] and algal pigment assemblage data (square-root transformation) through time in a reduced two-dimensional space (*SI Appendix*, Figs. S5 and S9). Sequential *t* test algorithm based on weighted mean values of stable states with prewhitening (*P* < 0.05, cut-off length of 5 to 15) and calculation of cumulative sum of differences were performed on major paleoenvironmental time series, to identify significant tipping points, thresholds, and potential regime shifts of the century-long biophysical processes (67). The detected sedimentary paleovariables include mass MS, Zr/Rb ratio, pollution load index of trace metal (Cd, Pb, Sb) particularly from atmospheric deposition (*SI Appendix, Text S2*), chlorophylls (sum of chlorophyll-a and pheophytin-a), pigments inferred UVR index, total *sed*aDNA concentration, and the numbers of taxa and DNA sequence reads of terrestrial plants.

### RoC analysis.

To estimate the RoCs of biophysical, socioeconomic, and climate variables and their relationships, we performed generalized additive model (GAM) analyses using z-score normalization of the univariate data time series. This is a well-validated and widely used method proposed by Wood (66) and developed by Simpson (31). The functions *gam* and *predict.gam* were used to choose the best fit models in R software (66), and GAM parameterization was performed following technical recommendations from ref. 31. All data series were z-score normalized before the first derivative simulation for RoC (*SI Appendix*, Fig. S11). To account for temporal autocorrelation, all models contained a continuous-time first-order autoregressive [CAR(1)] process. The RoCs were calculated based on 95% simultaneous CI generated from the posterior simulations of the fitted GAMs using the first derivative against time (31). This was achieved using the derivative function with 10,000 simulations of finite distance of 1e−07. The periods of significant dynamic change were identified where the simultaneous CI of the first derivative of the GAM function does not include 0 (31). For all fitted GAM analyses, the correlations with *P*-values ≤ 0.05 were considered significant. Diagnostic plots (i.e., first derivatives vs. time plots, observed values vs. fitted values plots) were visualized along with statistical summary to check for the robustness of the performed GAMs (*SI Appendix*, Figs. S11–S13 and Tables S1 and S2). Integrated RoC indexes for the subsystems were produced using entropy-weighted integration of RoC data series at the annual resolution. Where the simulation value does not include 0, the models detect increase or decrease in the RoCs for the system variables.

In this study, we synthesized multiple paleolimnological proxy records to determine the dominant ecological variability (i.e., RoC) of the Taihu lake-watershed system, including: i) frequency-dependent mass MS—soil erosion/stability; ii) total *sed*aDNA concentration – DNA/biomass production; iii) chlorophylls concentration (sum of chlorophyll-a and pheophytin-a)—lake primary production; iv) NMDS score of pigment assemblages—aquatic algae community structure (turnover); v) NMDS score of terrestrial plant *sed*aDNA—terrestrial plant community structure (see further description in Fig. 3 and *SI Appendix, Text S5* and Table S3) (17). Socioeconomic development status was synthesized from multiple social, agricultural, and industrial indicators including human population, rice yield, GDP per capita, and industry output (32). Taken together, we assume that ecoenvironmental degradation in the integrated SES can be tracked by increases in anthropogenic (mostly land-use driven) soil erosion, primary production, and community turnover, whereas socioeconomic development is characterized by population explosion, agricultural intensification, and economic growth (17, 26).

### Integrated RoC index.

Integrated RoC index for ecological degradation and socioeconomic development was produced separately, using the additive weighting method, to compress multisource data series into a simple measure (26, 32). We applied a classic entropy method (68), which is objectively based on the information entropy and variations in the selected indicators (69), to determine the weight of each individual ecological and socioeconomic variables. Then, the weighted curves of indicators were combined additively to produce two integrated RoC indices for biophysical process and socioeconomy, respectively. More specifically, we use five steps to conduct the entropy weighing analysis, namely data standardization, calculation of indicator proportion, calculation of information entropy, calculation of entropy redundancy, and finally weight determination (Eqs. **1**–**5**). Detailed calculations are as follows.

Data standardization to eliminate the influence of dimension and magnitude for the indicator *j* in year *i*

[1] $$X_{ij}^{'}=\frac{\left(X_{ij}-\min\left\{X_{j}\right\}\right)}{\left(\max\left\{X_{j}\right\}-\min\left\{X_{j}\right\}\right)}$$

The proportion of the indicator *j* in year *i*

[2] $$Y_{\mathrm{ij}}=X_{\mathrm{ij}}^{'}/\sum_{i=1}^{m}X_{\mathrm{ij}}^{'}$$

Information entropy of the indicator *j* across year *m*

[3] $$e_{j}=-\frac{1}{\ln\;m}\sum_{i=1}^{m}Y_{\mathrm{ij}}\times \ln\;Y_{\mathrm{ij}}\;(0\leq e_{j}\leq 1)$$

Entropy redundancy of the indicator *j*

[4] $$d_{j}=1-e_{j}$$

Weight of the indicator *j*

[5] $$w_{j}=d_{j}/\sum_{j=1}^{n}d_{j}$$

Weighted result of an integrated RoC index in year *i*

[6] $$S_{i}=\sum_{j=1}^{n}w_{j}\times X_{\mathrm{ij}}$$

where *m* denotes total years, and *n* is the total number of indicators; $X_{\mathrm{ij}}$ represents the RoC value of indicator *j* in year *i*, $X_{\mathrm{ij}}^{\prime }$ is the 0 to 1 normalized value, and min $\left\{X_{j}\right\}$ and max $\left\{X_{j}\right\}$ indicate the minimum and maximum values of indicator *j* among all years, respectively; $Y_{\mathrm{ij}}$ presents the proportion of the indicator *j* in year *i*; $e_{j}$ is the information entropy of indicator *j*, $d_{j}$ is the entropy redundancy of indicator *j*, and $w_{j}$ indicates weight of the indicator *j*; $S_{i}$ represents the weighted and additive result of an integrated RoC index for ecological or socioeconomic system in year *i*.

### Coupling model.

Coupling depicts the phenomenon by which two or more systems affect each other through interactive mechanisms (32, 43, 46). This concept has been widely applied in the areas of climate or environmental studies, however, it has rarely been used in long-term, intertwined social–ecological research (6, 70). To further understand the dynamic connections and feedback between human and natural subsystems in the interlinked Lake Taihu social–ecological system, we applied a RoC-based numerical model to estimate their dynamic coupling degree, namely the CI (10). CI here refers to the ratio of i) change in the rate of ecosystem degradation ($S_{\mathrm{iE}}$) and ii) change in the rate of socioeconomic development ($S_{\mathrm{iS}}$) during a certain period of time (year *i*), which can be calculated as

[7] $$CI=S_{\mathrm{iE}}/S_{\mathrm{iS}}$$

As a quantitative evaluation criterion of the SES transient dynamics, when CI ≥ 1, it means that the rate of ecoenvironmental degradation keeps pace with or is higher than socioeconomic development within an unsustainable system; when CI = 1, it is the turning point between absolute coupling and relative decoupling; when 0 < CI < 1, it means that the rate of ecoenvironmental degradation falls short of that of socioeconomic development within a sustainable system; when CI = 0, it means the socioeconomy is developing, while ecological status remains stable; when ecological status improves while the socioeconomy keeps developing in a desirable and more sustainable system, then CI < 0.

### Ecological footprint.

The EF is a powerful measure of the demand that human populations and activities place on the biosphere in a given year, considering the prevailing technology and resource management of that year (71). As a widely recognized measure of sustainability, the EF provides an integrated, multiscale approach to tracking the use and overuse of natural resources, and their consequent impacts on ecosystems. In this study, human consumption-based EF was used to further test the relationship between economic growth and the environment in recent decades (72). This EF is usually measured in global hectares (gha), which represents one hectare of biologically productive land at the global average productivity level in terms of six land-use types (73), namely cropland, grazing land, forest land, inland fishing grounds, built-up land, and fossil energy land (*SI Appendix*, Table S4 and S5). The basic model of EF is as follows.

[8] $$EF=N\times ef=N\times \sum (EQF_{i}\times C_{i}/P_{i})$$

where *EF* is the total ecological footprint, *N* is the human population, *ef* is the per capita ecological footprint, $EQF_{i}$ is the equivalence factor corresponding to the *i*th type of land (74), $C_{i}$ is the per capita consumption of the *i*th land, and $P_{i}$ is the global average production capacity of the *i*th land (74, 75).

Given the data availability, we calculated the regional EF of Jiangsu Province (including cities of Wuxi, Changzhou, and Suzhou surrounding Lake Taihu, Fig. 2) during the period of 1980 to 2020, using consumption data from the Provincial Statistical Yearbooks (http://tj.jiangsu.gov.cn/) and the National Bureau of Statistics of China. For comparison, the national EF time series of China was also obtained from the National Footprint Accounts dataset (74) (*SI Appendix*, Fig. S15).

Agricultural development and urbanization also profoundly influence the use of water resources (8, 41). Considering this issue, a separate water resource EF was calculated for Lake Taihu watershed to reflect the amount of freshwater required for various human activities and its environmental effects (*SI Appendix*, Table S4).

## Supplementary Material

Appendix 01 (PDF)

## Data Availability

Data that support the findings of this study are included in the article and its *SI Appendix*. Original data investigated in this work are available through Mendeley Data repository (76). The code used for the analysis is openly available in R software based on the original literature (31, 65, 66).
