# Supplementary material for: Transient social–ecological dynamics reveal signals of decoupling in a highly disturbed Anthropocene landscape
Source: Proc Natl Acad Sci U S A. 2024 Apr 19;121(17):e2321303121. doi: 10.1073/pnas.2321303121 (PMC11046650; doi:10.1073/pnas.2321303121)
Supplement: Supplementary file 1 — Appendix 01 (PDF) [file pnas.2321303121.sapp.pdf]

PNAS

Supplementary Information for

Transient social-ecological dynamics reveal signals of decoupling in a highly disturbed Anthropocene landscape

Qi Lin[1], Ke Zhang[1,2], Charline Giguet-Covex, Fabien Arnaud, Suzanne McGowan, Ludovic Gielly, Eric Capo, Shixin Huang, Gentile Francesco Ficetolad, Ji Shen[2], John A. Dearing, and Michael E. Meadows

[1]Q.L. and K.Z. contributed equally to this work.

[2]Correspondence: Ke Zhang or Ji Shen
Email: kzhang@niglas.ac.cn or jishen@nju.edu.cn

This PDF file includes:

Supplementary Text S1 to S6
Supplementary Figures S1 to S15
Supplementary Tables S1 to S5
SI References

Text S1. Recent climate change and socioeconomic development

Lake Taihu region is dominated by a subtropical monsoon climate. According to the instrumental records from 1950 to 2020 CE (*SI Appendix*, Fig. S11), the annual mean air temperature was 16.3±0.6 (SD) ℃, the annual precipitation was 1172.3±210.3 mm, and the annual evaporation was ~1001 mm. Notably, the region had a long-term warming trend over the past 70 years (linear fitting R^2=0.42, p<0.01), and the annual mean temperature has risen by ~1.8 ℃. Since 1980 CE, the temperature increase was even more pronounced (R^2=0.61, p<0.01). The annual precipitation and monthly anomalies did not show a long-term trend. Instead, high precipitation occurred during the periods of 1950–1960, 1980–2000 and after 2008 CE. Correspondingly, water level gauges recorded high frequency floods occurring around the 1950s and 1980s–1990s.

The region has several thousand years of history of agricultural activities, and in recent decades, it has become one of the most densely populated and rapidly developing regions of China (1, 2). In Wuxi city of the northern watershed (upper), for example, the population have continuously grown ~2-fold to 4.8 millions over the past half-century; meanwhile, the GDP had an growth rate of 16±12%, with the highest growth period of 1980–2000 CE (*SI Appendix*, Fig. S10). Rapid population and economic growth have dramatically changed land use and land cover in the watershed, which was characterized by fast expansion of urbanized land at cost of a massive loss of cropland. The urbanized areas in the 40 km-radius buffer increased by ~11 times in 2010 CE compared to the 1980s (*SI Appendix*, Fig. S2). Still, the land cover is dominated by cropland (41.1%), followed by urbanized land (18.7%). Document records showed recent century-long decreasing trend of cultivated land area in Wuxi city, but the grain production continued to rise since the 1950s, and fell down after 2000 CE. Crops are dominated by *Oryza sativa*, *Triticum aestivum*, *Brassica napus*, *Gossypium*, and leguminous plants, along with small area of orchard plantations. Woodland vegetation is mainly distributed in the southwestern hilly areas, including mixed *Pinus massoniana,* deciduous *Quercus*, and evergreen *Castanopsis* types etc., with some distributed around the northern lakeshore dominated by deciduous coniferous species. Grassland and wetland surrounding the lake are dominated by various species of Poaceae, and *Cyperaceae* and *Polygonum*.

Text S2. Traditional sediment proxy analysis

Physical proxy. Sediment grain size analysis was performed using a Malvern Instrument Mastersizer-2000 after treating ~1 g of well-mixed freeze-dried sample with 5% H_2O_2 (~24 hours) to remove organic matter and 10% HCl (~24 hours) to remove carbonates at room temperature, washing with distilled water to near-neutral pH solution, and ultrasonic dispersal with $(NaPO_3)_6$. Instrumental accuracy is based on polymer microsphere standard, series 4000, provided by Duke Scientific Corporation. In seven measurements over three years all median grains size results were <1.2% from the stated standard value of 0.993 mm. Three repeated measures of the sediment samples indicated high measurement repeatability and stability, with maximum standard deviation of median diameter <0.3% in the samples. Mass specific low frequency magnetic susceptibility (MS) of the sediment samples was measured at 0.47 kHz using a Bartington MS2 Meter (3).

Nutrient and trace metal. Total organic carbon (TOC) and total nitrogen (TN) content in the samples, ground and pre-treated with 1 mol L^{-1} HCl at room temperature to remove carbonate, was measured using a Euro 3000 elemental analyzer. The results are expressed in % of dry weight sediment. Standard reference materials GSS-9, GSS-13 and GSS-16 (lacustrine sediment and soil from National Institute of Metrology of China) were used as a quality control, and the measured values were all within the ranges of certified values. After sequential digestion of the freeze-dried, ground samples (4), concentrations of sediment total phosphorus (TP) were determined by inductively coupled plasma-atomic emission spectrometry (ICP-AES; Leeman Labs, Profile DV), and concentrations of trace metals (such as Cd, Pb, Cd) were determined by inductively coupled plasma mass spectrometry (ICP-MS; Agilent 7700x). These results are expressed in mg kg^{-1} dry weight sediment. The data quality was ensured by measuring blanks and standard reference materials (GBW07358; Chinese geological reference materials) after every tenth sample.

Contamination assessment. Pollution load index (PLI) is widely applied to assess total level of trace metal contamination (4). Mathematically, PLI is calculated as $PLI = (CF_1 \times CF_2 \times CF_3 \times \cdots \times CF_n)^{1/n}$; $CF = [M]_{sample} / [M]_{background}$. Where CF is the ratio obtained by dividing the concentration of each metal in the sediment by the background value. The bottom sediments (n=2) of cores from Taihu Lake, which were deposited in pre-industrial periods (i.e. before the 1920s), could be considered as reference background (3, 4). Pollution load index (PLI) of Cd, Pb, and Sb was thus determined to reflect anthropogenic pollution particularly from atmospheric deposition (3, 4). When PLI>1, it means that trace metal contamination exists.

Subfossil pollen. Plant pollen analysis was conducted on ~2 g of dried sub-samples following ref. (5). One tablet of Lycopodium spores (27,637 ± 563 grains/tablet) was added to each sample as a tracer for calculating the pollen concentration. All samples were treated with 10% HCl, 10% NaOH, and 2.1 g cm^{-3} heavy liquid flotation (the conventional HF method was not used due to the lack of siliceous materials in the sediment) before being sieved through a 7 μm mesh and acetolyzed. Finally, the samples were preserved in glycerine. All pollen and spores were identified under ×400 magnification using an Olympus BX-53 microscope to a sum of over 500 grains. Aquatic taxa were removed, and the remaining terrestrial taxa were expressed as percentages of the sum of the terrestrial pollen assemblages. The pollen concentrations of major vegetation taxa were calculated as grains g^{-1}.

Pigment biomakers. Sedimentary chlorophyll and carotenoid pigments were analyzed using an Agilent 1200 series HPLC (Agilent Technologies, Mississauga, ON, Canada) with quaternary pump, autosampler, ODS Hypersil column (250×4.6 mm; 5 μm particle size), and photo-diode array detector as described in ref. (6) with the extraction and separation conditions modified from ref. (7). Pigments were calibrated using authentic standards (DHI, Denmark) and are expressed in nmol pigment g^{-1} TOC to compensate for diagenetic degradation (8). The ultraviolet radiation (UVR)-absorbing pigment (scytonemin derivative) identified in ref. (9) was divided by the sum of key carotenoids (diatoxanthin, lutein-zeaxanthin and alloxanthin) and multiplied by 100 to derive a UVR index. Calibration in whole-lake experiments revealed that this index increases as a linear

function of the depth of UVR penetration, such that higher index values indicate greater exposure to potentially damaging UVR (9), namely better light conditions and indicate higher water clarity (10).

Text S3. Sedimentology and geochemical characteristics

Major physicochemical characteristics of the core sediments were analyzed, and the results are presented in Fig. 2 and *SI Appendix*, Figs. S3 and S7. Color of the sediments varied from gray to dark-brown from the bottom to the upper core, and then appeared grayish yellow in the top centimeters. Water content of the sediments was 44.4±4.2%. The sediments were mainly composed of silt (4–63 μm) and clay (<4 μm), accounting for 81.6±1.7% and 17.4±2.0%, respectively. The median grain size was 11.7±1.4 μm, and showed an overall decreasing trend in contrast to increasing fine silt and clay contents from the bottom to the top of the core. The value of mass magnetic susceptibility (MS) ranged between 16×10^{-8}–27×10^{-8} m^3 kg^{-1}, showing a sharp increase in the 1960s and maintaining high values afterward. Total organic carbon content in the sediments was 0.87±0.21% with an increasing trend, whereas C/N ratios showed a decreasing trend that is generally less than 10. Sediment TP increased significantly from the middle of the core (about the 1980s), and showed a decrease in the top centimeters. Based on the XRF scanning results, zirconium and zirconium-to-rubidium (Zr/Rb) of the core sediments showed an sustained increase during 1900–1950 CE and abnormally peaks during 1950–1980 CE, followed by a significant decrease.

Text S4. Plant *seda*DNA records

Fluorescent quantification results showed that total DNA concentration in the 23 slices of sediments fluctuated largely between 1.09–98.11 ng g^{-1} with an average of 23.88±29.91 ng g^{-1} (Fig. 3). After high-throughput sequencing and standard filtering procedure, we obtained 1,920,292 usable merged reads, corresponding to 98 terrestrial sequences (including herb, shrub and tree taxa). Comparing them with the "Flora of China" (http://iplant.cn/foc) as listed in Jiangsu and Zhejiang provinces around the lake region, 57 well-identified taxa among 23 samples (from 3 to 55 taxa per sample) were retained and used to investigate watershed vegetation dynamics over the past hundred years (*SI Appendix*, Fig. S8). Temporally, total *seda*DNA concentration changed similarly with the plant DNA content and richness as indicated by the numbers of taxa and DNA reads (Fig. 3). Four phases of changes were recorded as follows: low level before the 1950s; slight increase during the 1950s–1980s; sharp increase until high level during the 1980s–2000s; and gradual decline after the 2000s. Nonmetric multidimensional scaling (NMDS) of plant DNA chronosequences (*SI Appendix*, Fig. S9), using the number of reads in four PCR replicates, showed higher replicate reproducibility during the 1980s–2000s when DNA quantity and number of taxa were high. This analysis of similarities confirms the significant difference of plant communities observed during the 1980s–2000s, and also proves the reliability of the *seda*DNA records (11).

Comparing the plant DNA results with modern vegetation compositions in the watershed (12), we found that the plant diversity recovered through *seda*DNA in the core clearly provides a relatively

local signal (Fig. 3 and *SI Appendix*, Fig. S8). Percent results of plant DNA reads showed that trees accounted for 18.0±7.7% with an obvious decrease during the 1950s–1960s in contrast to herbs, and crops accounted for 34.7±10.9% with several peaks in the 1920s, 1950s, 1970s–1990s and 2010s. We then focused on *sed*aDNA of major anthropochore taxa (13) to track the floristic dynamics associated with human activities, such as agriculture and urbanization. Temporal consistencies are recorded among Brassicaceae, Poaceae, *PACMAD* clade, *BOP* clade, and Hordeinae, probably indicating historical crop cultivation such as rapeseed and/or cabbage, and cereal taxa. Indeed, *PACMAD* and *BOP* clade reflect specific DNA information from most cereal crops including maize, millet, sorghum (panicoideae subfamily) and rice (oryzoideae subfamily), wheat, barley, oat and rye (pooideae subfamily), respectively. The temporal evolution of these taxa is in good agreement with the grain production data collected and showing a maximum between the 1960s and 2000s (Fig. 4). While the exact composition of the cereal crops is difficult to establish due to the low taxonomic resolution, the presence of rice is supported by the detection of other herbs related to paddy fields (*SI Appendix*, Fig. S8b–c).

Taxa from the Rosaceae family, other than *Potentilla sp*. and Colurieae, may represent the cultivation of fruit trees. This cultivation increased sharply from the 1990s onwards, which is consistent with the high detection of DNA from Rosaceae while that from *Potentilla sp.* dropped. Changes in Pinidae and other taxa suggested that trees were dominated by coniferous species – the fast-growing pioneer taxa until the 1960s. Subsequently, increases in Betulaceae (including *Alnus sp.*), *Taxus sp.*, *Broussonetia Papyrifera* mainly reflected anthropogenic plantation for dike protection and erosion control along the lakeshore and riverside, and possible timber supply for building and paper industry (14). Some arboreal taxa that were widely cultivated in gardens and streets as ornament, such as *Acer sp.*, Caprifoliaceae (including *Lonicera sp.*) and Spermatophyta (including Ginkgo) (13), first increased in the 1960s and then enriched around the 1980s, following the human population growth and development of urban areas. Herbaceous plants, like *Galium sp.*, *Scorzoneroides sp.*, *Ranunculus sp.*, *Maianthemu sp.*, *Ononis sp.*, *Teucrium sp.*, Polygonoideae and others were also detected from the 1960s, and become abundant during the 1980s–2000s (*SI Appendix*, Fig. S8d). Over the last two decades, however, *sed*aDNA of most plant taxa was at a low level or even undetectable.

Text S5. Implications of multi-proxy paleoenvironmental records

Lake act as a sink for material influx from the watershed, and therefore sediment records, are invaluable archives to evaluate past natural and anthropogenic modifications of terrestrial landscapes and aquatic ecosystems, in particular combining classical paleo-proxies and emerging ancient DNA indicators (15–17). For example, rubidium (Rb) and titanium (Ti) are mostly found in the fine-particle fraction and are geochemically conservative (18); zirconium (Zr) is enriched mainly in the silty-sandy fraction that contain heavy minerals such as zircon (19). Increases in Zr/Rb point thus to more detrital inputs from coarse-grained sources (20). Sediment magnetic susceptibility (MS) depend on the abundance of magnetic minerals and also trace metals (21), which are typically supplied to lakes via watershed soil erosion and anthropogenic emissions (1, 22). Lake sediments can also provide a long-term history of changes in natural and

human pollutant input (e.g., toxic metals), and the possibility to evaluate temporal patterns of anthropogenic pollution (2).

Total extracellular DNA preserved in sediments is closely related to both lake and watershed biomass from all functional groups and the supply of fine particles (23–25). In particular, extracellular plant DNA becomes protected against microbial degradation when adsorbed on clay particles, and transported to lakes by surface runoff and erosion processes (25–27). In Lake Taihu, DNA was present in all samples through the sediment core, and showed a decrease in the upper part of the core against the expected degradation pattern of DNA decrease with depth/time. However, in 5 samples corresponding to CE 1915, 1930, 1950, 2011 and 2018, both the number of DNA reads and of taxa are very low, which has to be considered with caution. Our comprehensive analyses of plant *sed*aDNA, pollen, and geochemical records, which were subdivided into four phases of changes (Figs. 3 and 4), can be used to unveil century-scale dynamic changes of surface erosion, pollution, vegetation, and their relationships with human landscape shaping in the watershed.

Meanwhile, autotrophic communities are key components in determining ecosystem state, and they are strong indicators of water ecological dynamics in lakes (28–31). Paleolimnological analysis of fossil photosynthetic pigments is an effective method to determine past ecological responses from nutrient enrichment and climate change, with different algal groups responding to different environmental drivers (4, 32). Sedimentary chlorophylls (e.g., chlorophyll-a and its chemically-stable production of degradation – pheophytin-a) represents the pigments from total (ubiquitous) algae, thus can indicate lake primary production. Considering potential degradation effects with depth/age (30–32), chlorophylls in the topmost core sediment are abnormally high thus often be excluded from the variability analysis. The UVR index inferred from pigments has been proven to be a good indicator for past water clarity (7, 8). Taken together, our analysis of sediment organic matter (TOC and C/N ratios), nutrients (TP), and particularly pigments can track past lake ecosystem dynamics.

Text S6. Research limitations and prospects

Although this study provides new methodological insights and inspiring findings, there are potential limitations and prospects that deserve increasing research investment. First, the metabarcoding approach developed here led to limited taxonomic resolution of plant taxa (especially cereal crops) we identified, which reduces the interpretative capacity. The development of a complete DNA reference library for local/regional species and the use of more specific primers (e.g., for Poaceae) (24, 33) is a key priority to fully exploit the power of metabarcoding for past, longer time-scale community reconstruction and paleoecological evolution. Second, taphonomic (e.g., poor DNA transfer or preservation) and other analytical (e.g., PCR inhibition) issues can also appear and led to misleading results (17, 25). However, in sedimentary systems highly controlled by detrital inputs, as in the case of Lake Taihu, we assume that taphonomic issues are reduced, although changes in the type of erosion (e.g., soil surface vs deep soil horizons) may affect the quality of the reconstructions (25, 26). Third, the identified critical transitions of SES were based on empirical records with entropy-weighted integration (see

the *Materials and Methods*), with associated caveats. Despite the enormous advantage of time series that *seda*DNA and other paleoenvironmental data provided, their availability, accuracy, and precision can always be improved (34), particularly through more robust age models, multi-proxy and/or multi-source data comparison and validation (e.g., monitored and surveyed data) (35). The weights of the indicators/variables for the SES synthesis were only calculated using the classical numerical method, while not considering their specific ecological or socioeconomic roles in the system, primarily because most of the indicated processes are either not necessarily mutually exclusive, or are difficult to quantify (15). Nevertheless, we believe the integration of *seda*DNA and other paleoenvironmental records in long-term social-ecological coupling research will lead to a more thorough understanding of Anthropocene environmental evolution in the Earth surface system and provide a more detailed picture of the complex mechanisms. As increasing data and modelling parameters become available in the future, the suggested approach can readily be applied to generate new knowledge for sustainable management of ecosystems, landscapes, and resources.

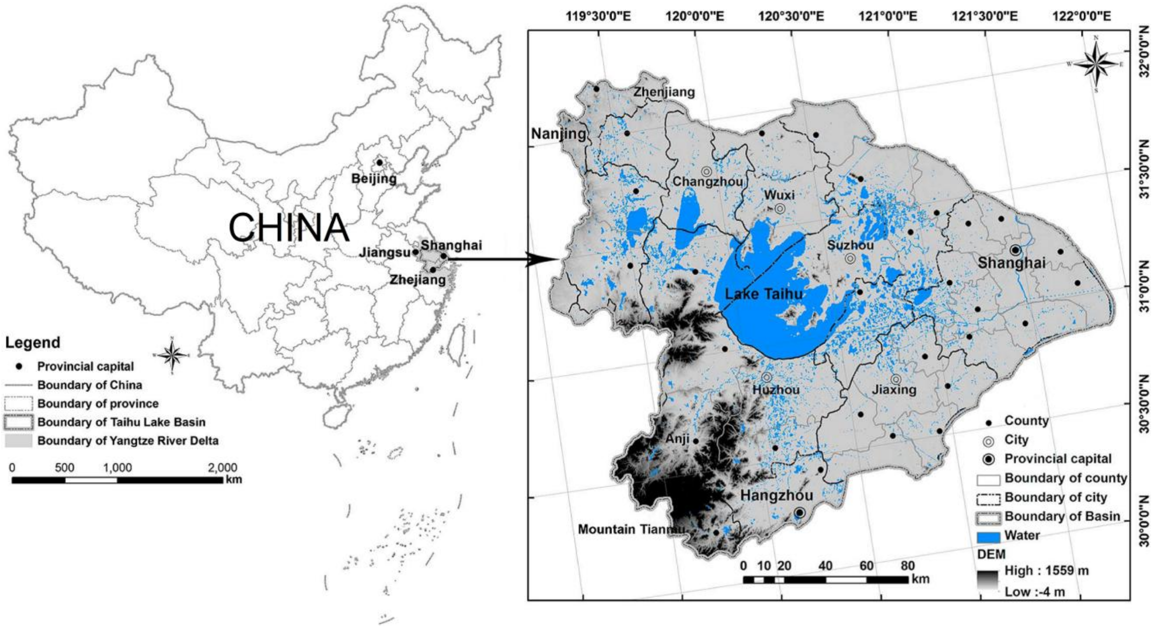

Fig. S1. Location of the Yangtze River Delta and Lake Taihu watershed in China.

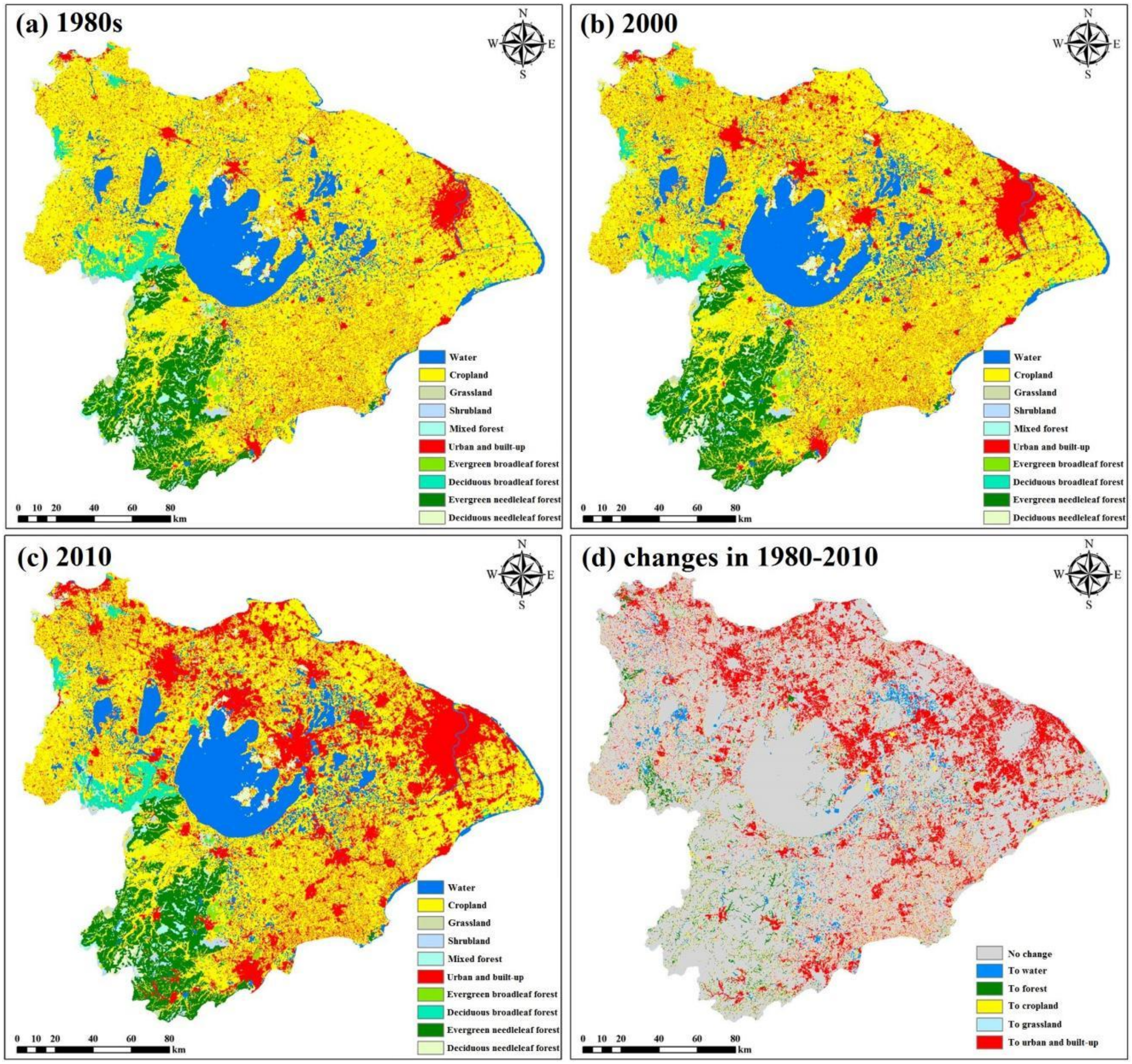

Fig. S2. Changes in land-use cover in the Lake Taihu watershed from the 1980s to 2010. Land-use data is interpreted from Landsat (TM/ETM+) images at the scale of 1:100,000, and can be accessed through the Lake and Watershed Data Center (https://lwdc.niglas.cas.cn).

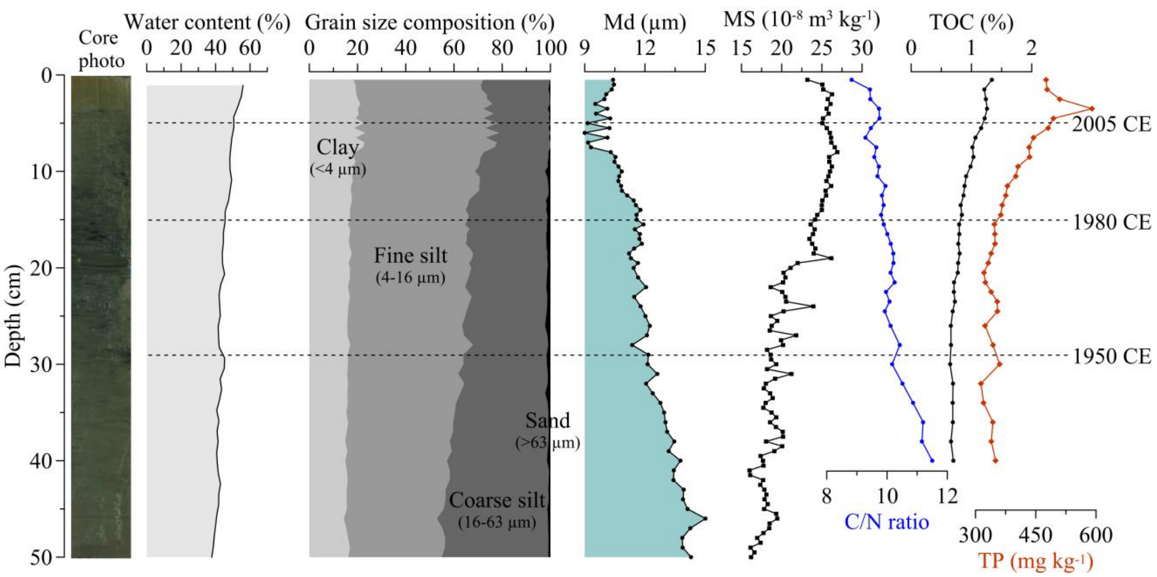

Fig. S3. Photograph of sediment core (TAI-18-2) from Lake Taihu, and vertical variations of sediment water content, grain size compositions, median grain size (Md), mass magnetic susceptibility (MS), total organic carbon (TOC), TOC/TN molar ratios (C/N), and total phosphorus (TP). Four general phases can be defined by the horizontal dashed lines.

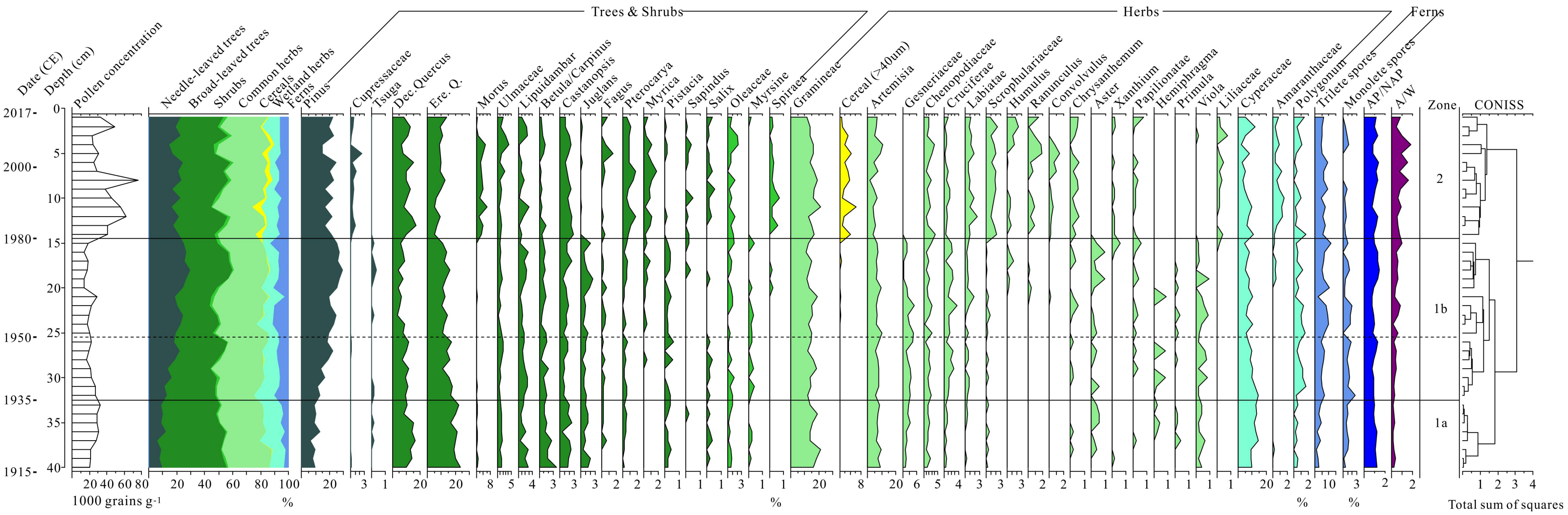

Fig. S4. Historical profiles of total terrestrial pollen concentrations, different pollen assemblages, and key pollen indicators related to major land cover change inferred from Lake Taihu sediments (TH1 core). The AP/NAP value (ratio of arboreal taxa proportion to non-arboreal taxa proportion) reflects forest variation. The A/W value(ratio of anthropochore taxa proportion to wetland taxa proportion) probably indicates the intensity of agricultural activities. Anthropochore taxa include plants which are spread by human activity and are intensively distributed in anthropogenic environments, such as *Xanthium*, *Humulus*, Chenopodiaceae, and other Compositae taxa. The wetland taxa in our study mainly include Cyperaceae and *Polygonum*. The sediment core is divided into two major pollen assemblage zones by CONISS (constrained incremental sum) clustering embedded in the Tilia program (36), with zone 1 containing two sub-zones (zone 1a and 1b).

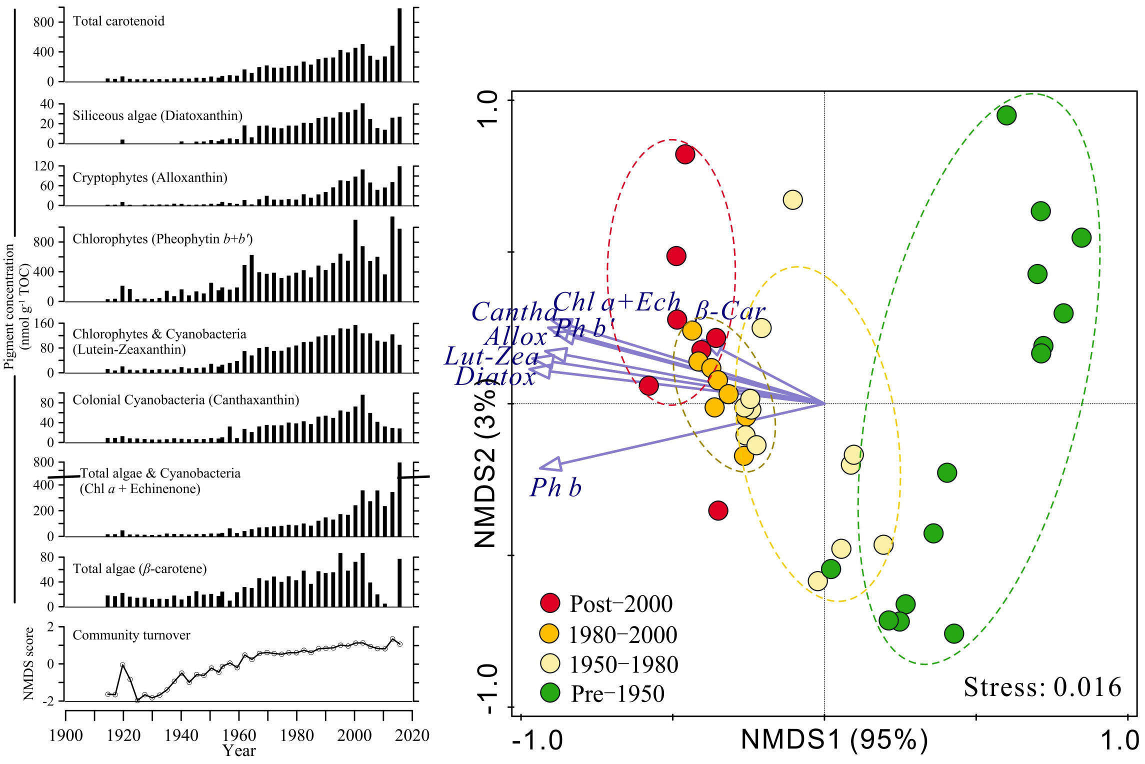

Fig. S5. Historical changes in algal chlorophyll and carotenoid pigments inferred from Lake Taihu sediments (TH1 core) (left), and nonmetric multidimensional scaling analysis (NMDS) of the pigment assemblage compositions (right). The dominant NMDS score (i.e., NMDS1 that explains 95% of variance) reflects algal community turnover. Dotted circles indicate the clustering groups as determined by the CONISS analysis.

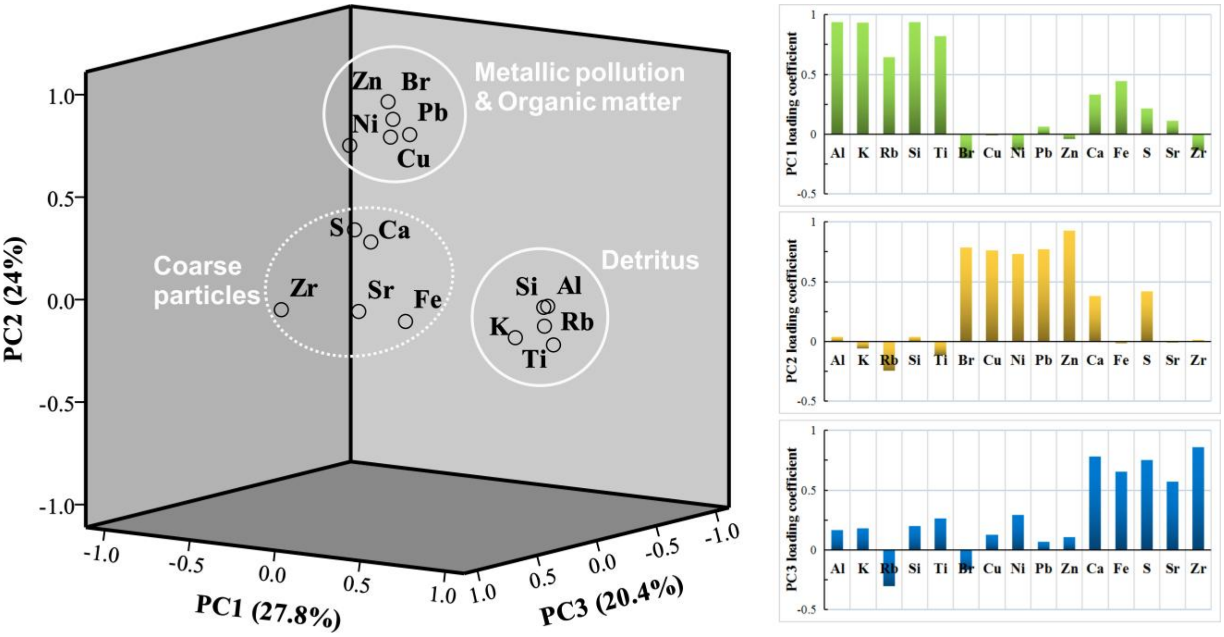

Fig. S6. Multivariate geochemical elements from XRF scanning of THM-2 sediment core and individual factor maps for the principal component analysis (PCA). The data can be transformed into three end-members (detritus, metallic pollution & organic matter, and coarse particles), according to their PC loading coefficients and geochemical properties.

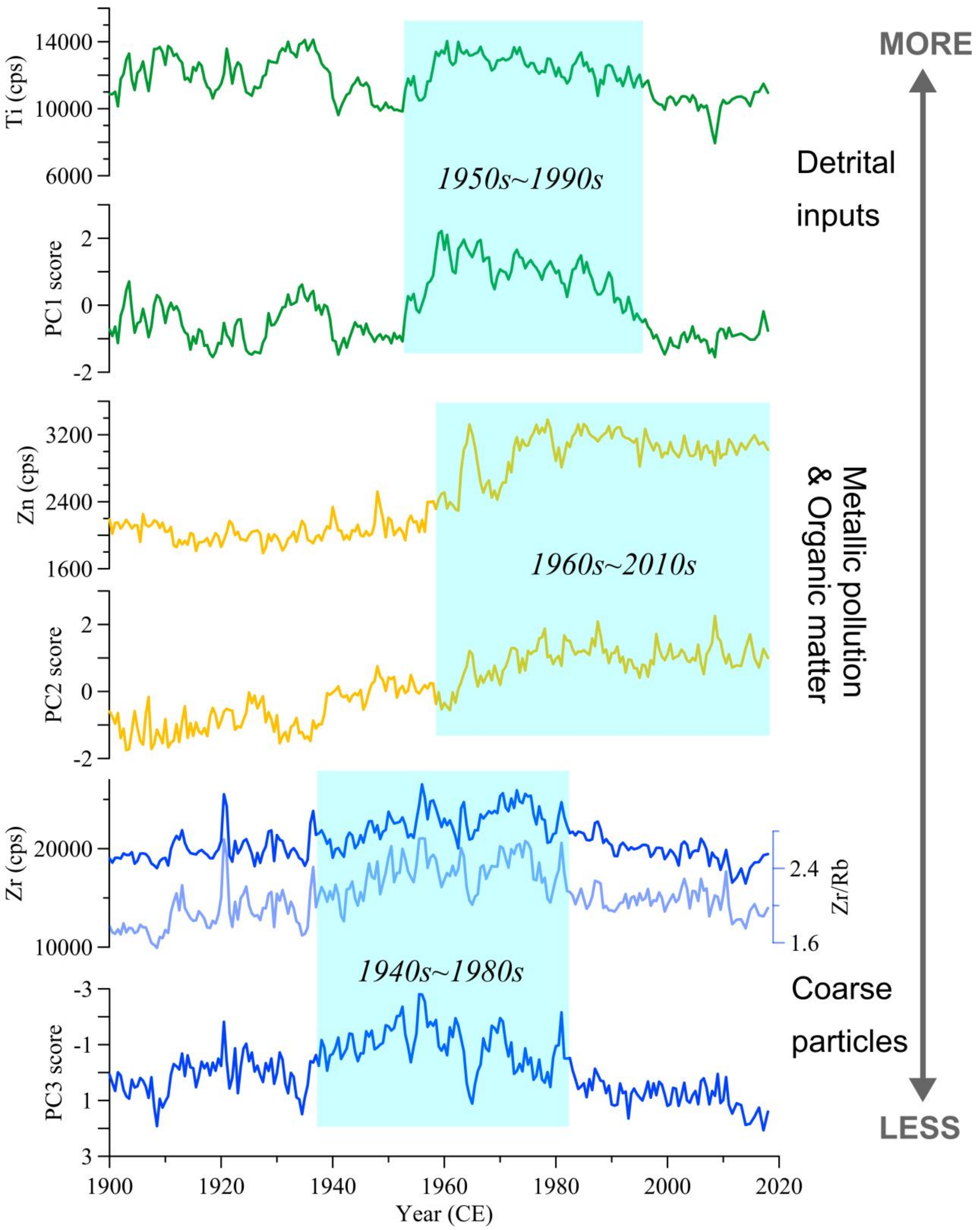

Fig. S7. Historical changes in typical geochemical elements from XRF results and three PC scores from the PCA results, together with their sedimentological and paleoenvironmental implications. Shaded zones shows periods of the relative high values over the past hundred years.

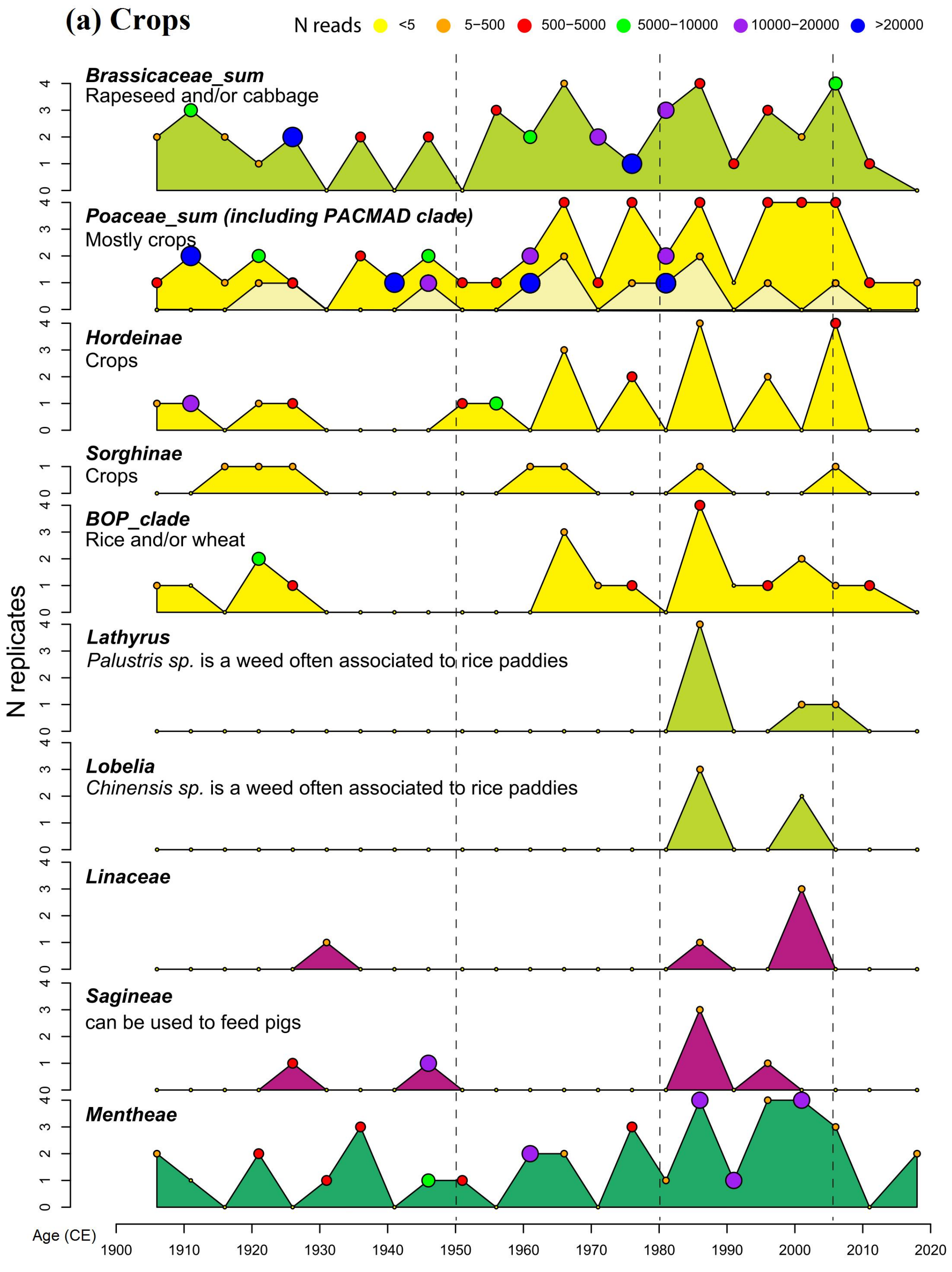
(a) Crops
N reads
<5
5−500
500−5000
5000−10000
10000−20000
>20000
Brassicaceae_sum
Rapeseed and/or cabbage
Poaceae_sum (including PACMAD clade)
Mostly crops
Hordeinae
Crops
Sorghinae
Crops
BOP_clade
Rice and/or wheat
Lathyrus
Palustris sp. is a weed often associated to rice paddies
Lobelia
Chinensis sp. is a weed often associated to rice paddies
Linaceae
Sagineae
can be used to feed pigs
Mentheae
N replicates
Age (CE)
1900
1910
1920
1930
1940
1950
1960
1970
1980
1990
2000
2010
2020

249

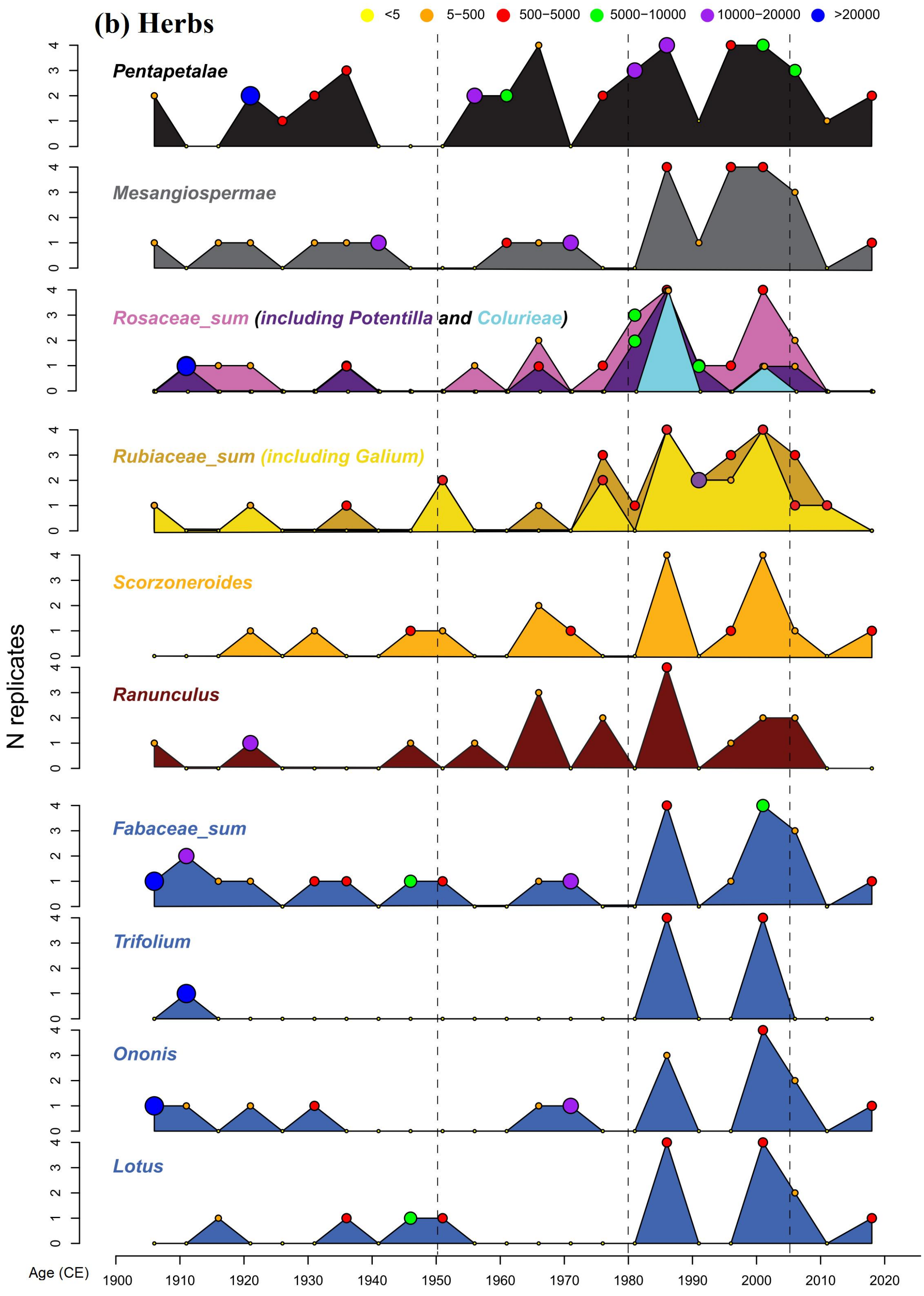
(b) Herbs
<5
5–500
500–5000
5000–10000
10000–20000
>20000
Pentapetalae
Mesangiospermae
Rosaceae_sum (including Potentilla and Colurieae)
Rubiaceae_sum (including Galium)
Scorzoneroides
Ranunculus
Fabaceae_sum
Trifolium
Ononis
Lotus
N replicates
Age (CE)
1900
1910
1920
1930
1940
1950
1960
1970
1980
1990
2000
2010
2020

251

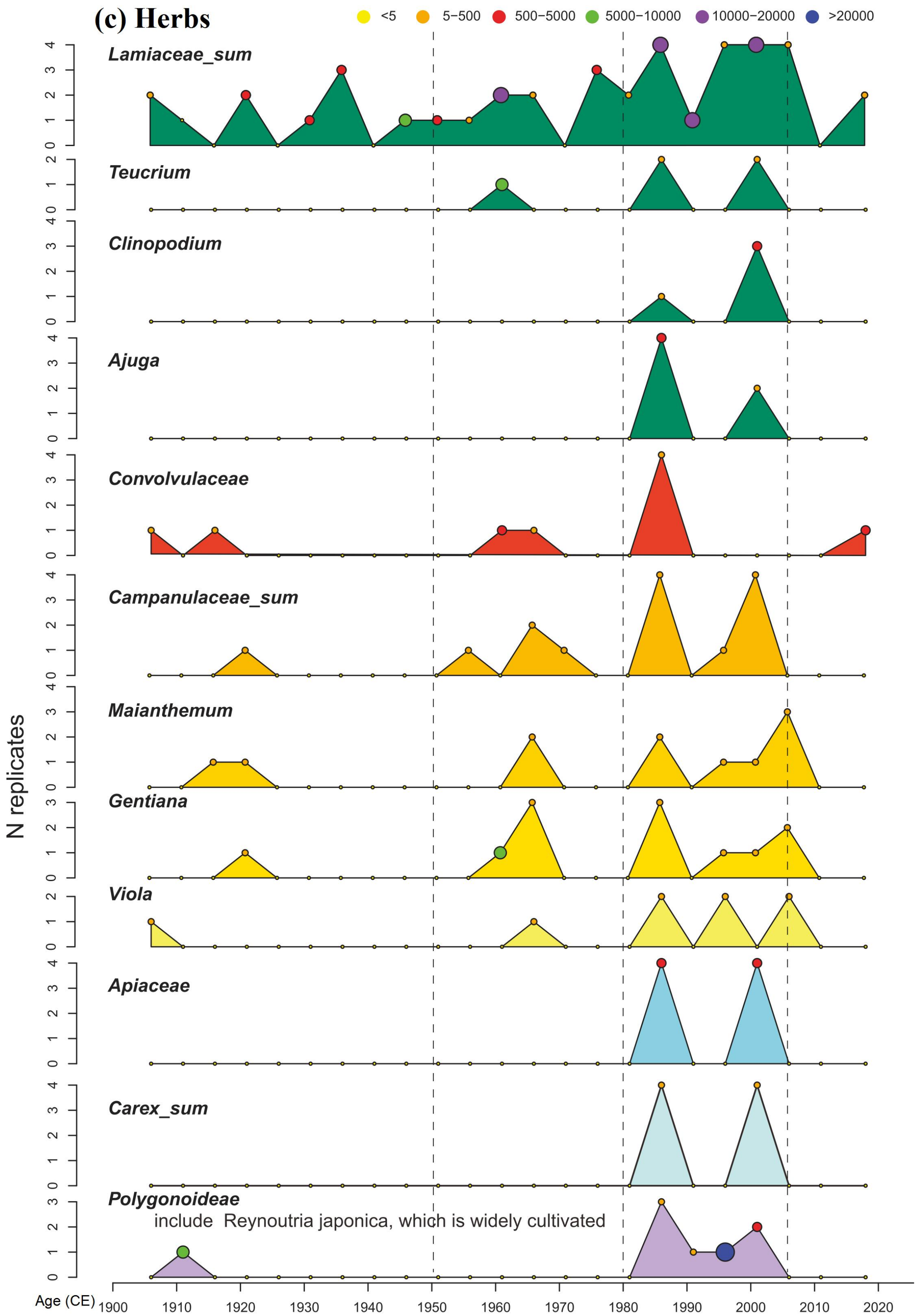

(c) Herbs
<5
5−500
500−5000
5000−10000
10000−20000
>20000
Lamiaceae_sum
Teucrium
Clinopodium
Ajuga
Convolvulaceae
Campanulaceae_sum
Maianthemum
Gentiana
Viola
Apiaceae
Carex_sum
Polygonoideae
include Reynoutria japonica, which is widely cultivated
N replicates
Age (CE)
1900
1910
1920
1930
1940
1950
1960
1970
1980
1990
2000
2010
2020

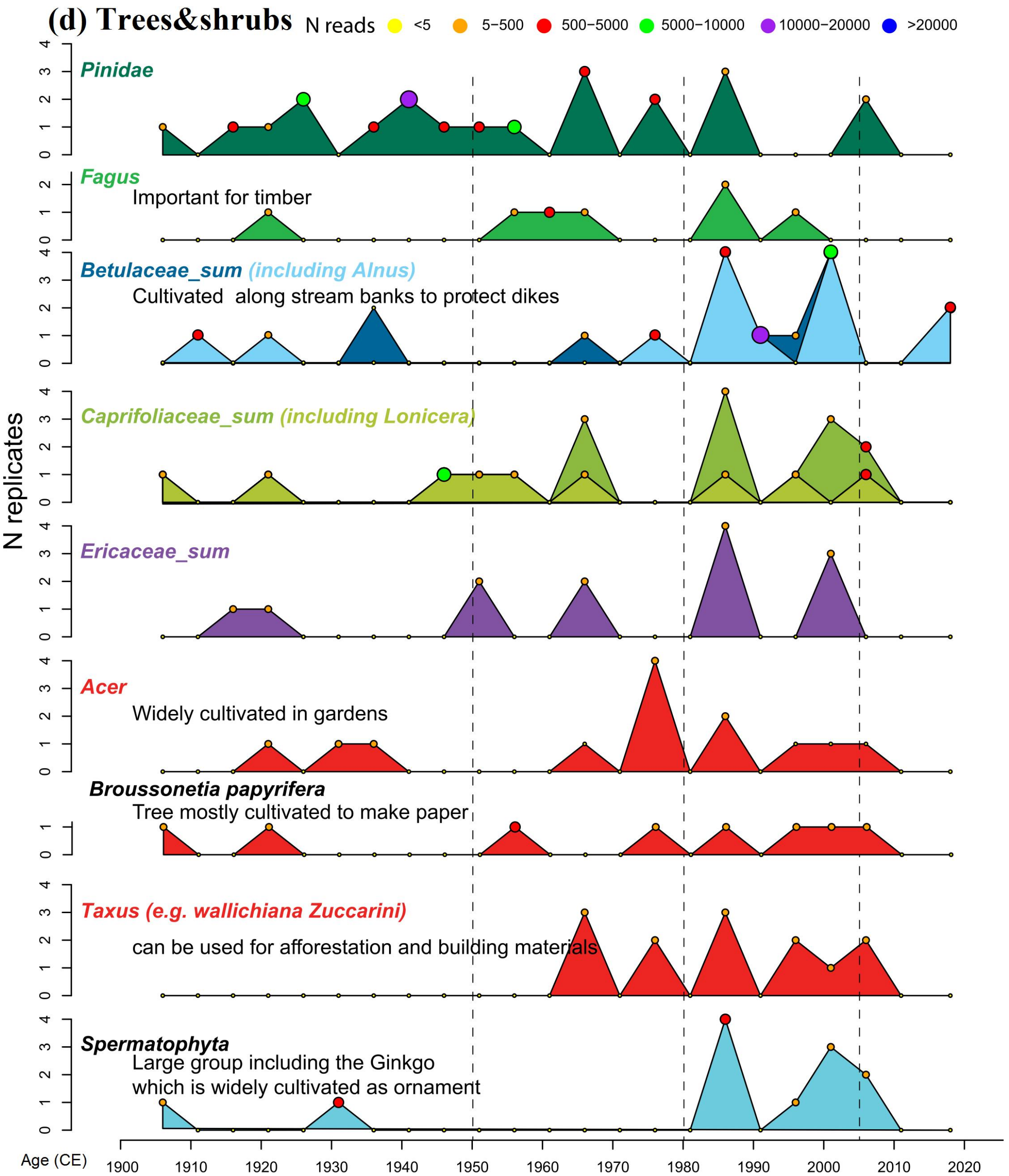

Fig. S8. Time series in community composition of terrestrial plants (a–crops; b, c–other herbs; d–trees & shrubs) inferred from the *sed*aDNA from Lake Taihu (TAI-18-2 core). The taxa in different groups are presented using the numbers of positive replicates (values on y-axis) and reads (size of circles on the top of the graphs). Potential interpretation of plant *sed*aDNA was shown along with some taxa strongly related to human activities. Four specific phases of changes in plant *sed*aDNA are defined by vertical dashed lines.

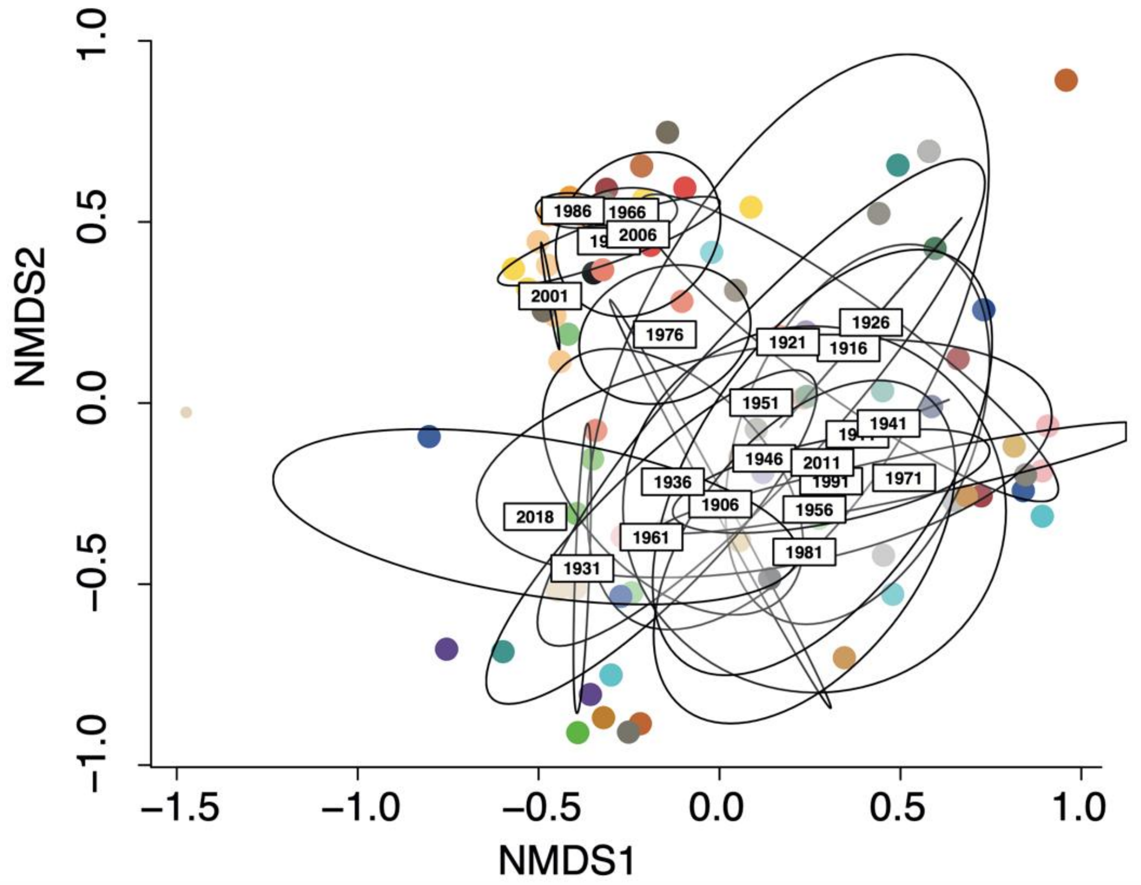

Fig. S9. Nonmetric multidimensional scaling (NMDS) analysis of plant *seda*DNA chronosequences based on the number of reads in four PCR replicates per sample.

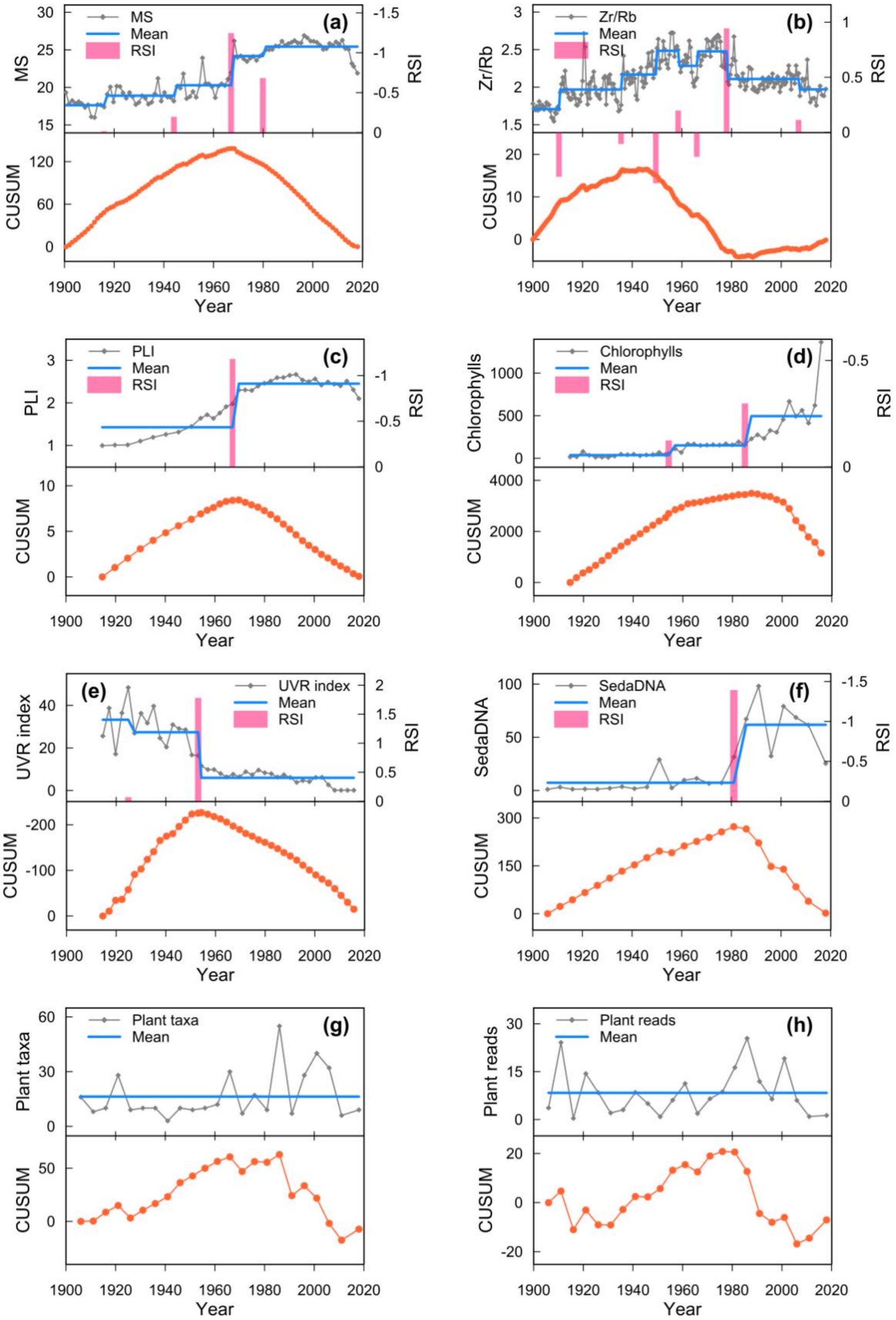

Fig. S10. Tipping points and potential regime shifts of the century-long biophysical processes in the complex system, as identified by sequential T-test algorithm based on segmental mean and calculation of cumulative sum of differences (CUSUM). The detected sedimentary paleo-variables include mass magnetic susceptibility (MS), Zr/Rb ratio, pollution load index of atmospheric trace metal (PLI), chlorophylls (sum of chlorophyll-a and pheophytin-a), UVR index, total *seda*DNA concentration, and the numbers of taxa and DNA reads of terrestrial plants. The Regime Shift Index (RSI) and CUSUM peaks indicate major shifting timings during the 1950s–1980s.

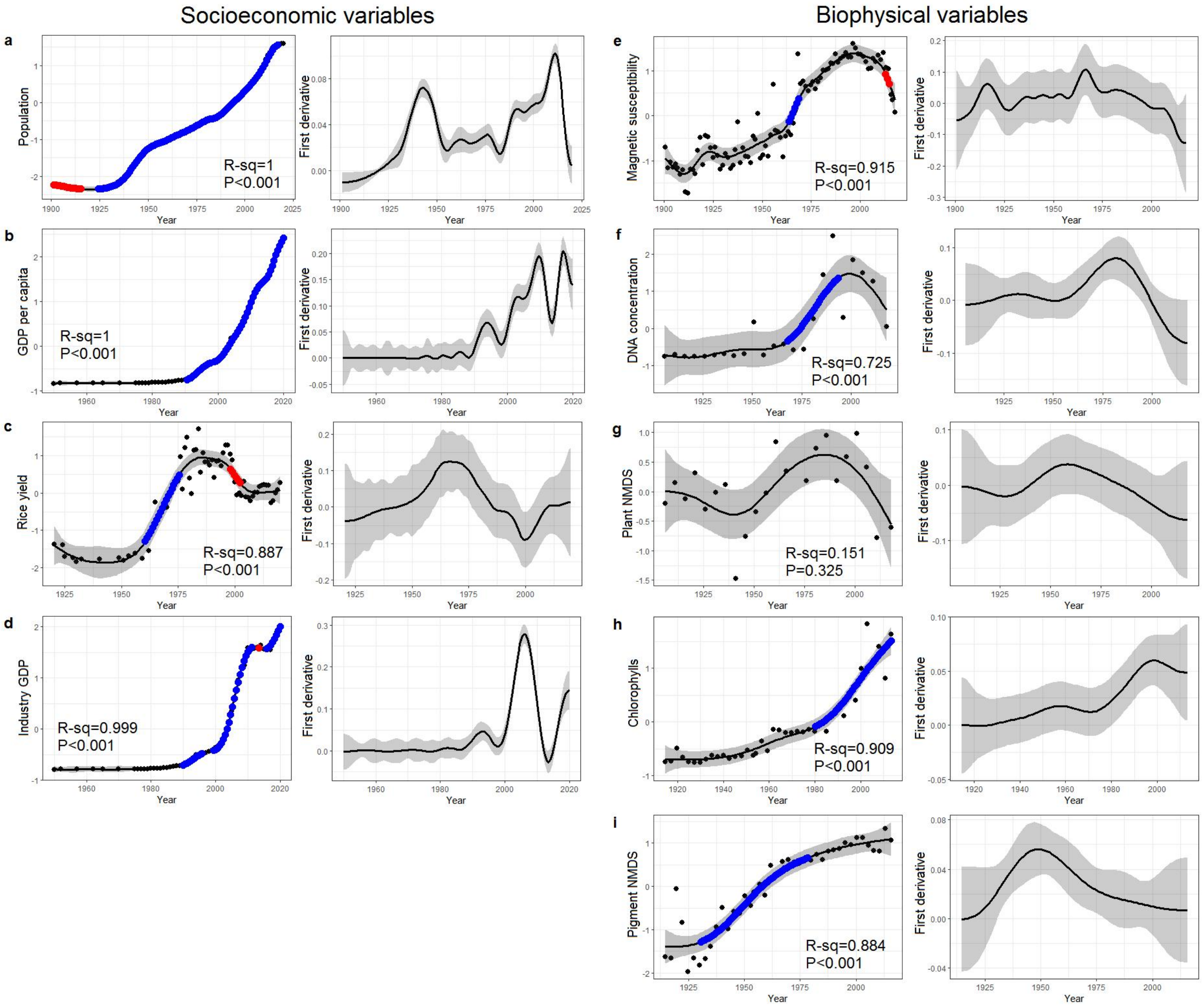

Fig. S11. GAM-based trends fitted to key socioeconomic (a–d) and biophysical (e–i) variables (z-score normalized) of Lake Taihu social-ecological system through time, and estimated first derivatives (black lines) with 95% simultaneous confidence intervals. The bold blue and red represents the significant periods of increase and decrease in the rates of change (RoCs) in the SES variables, respectively. Statistical summary for the GAMs is shown in *SI Appendix*, Table S1. GAM, generalized additives model.

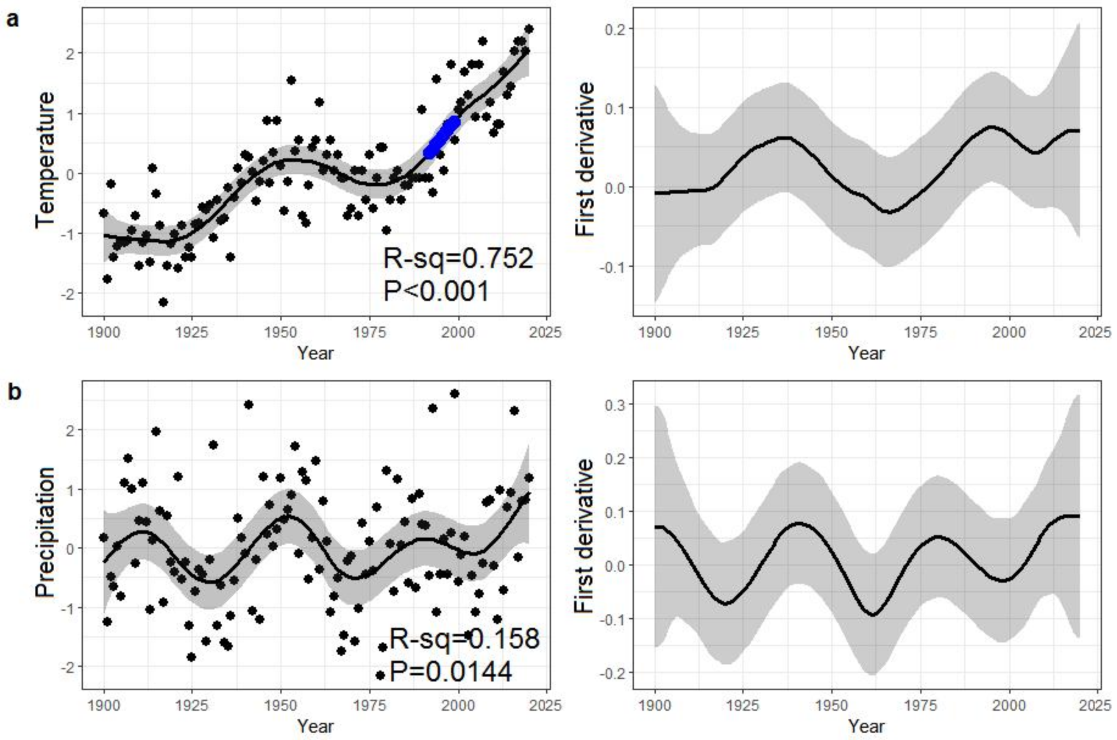

Fig. S12. GAM-based trends fitted to climate variables (z-score normalized) through time.

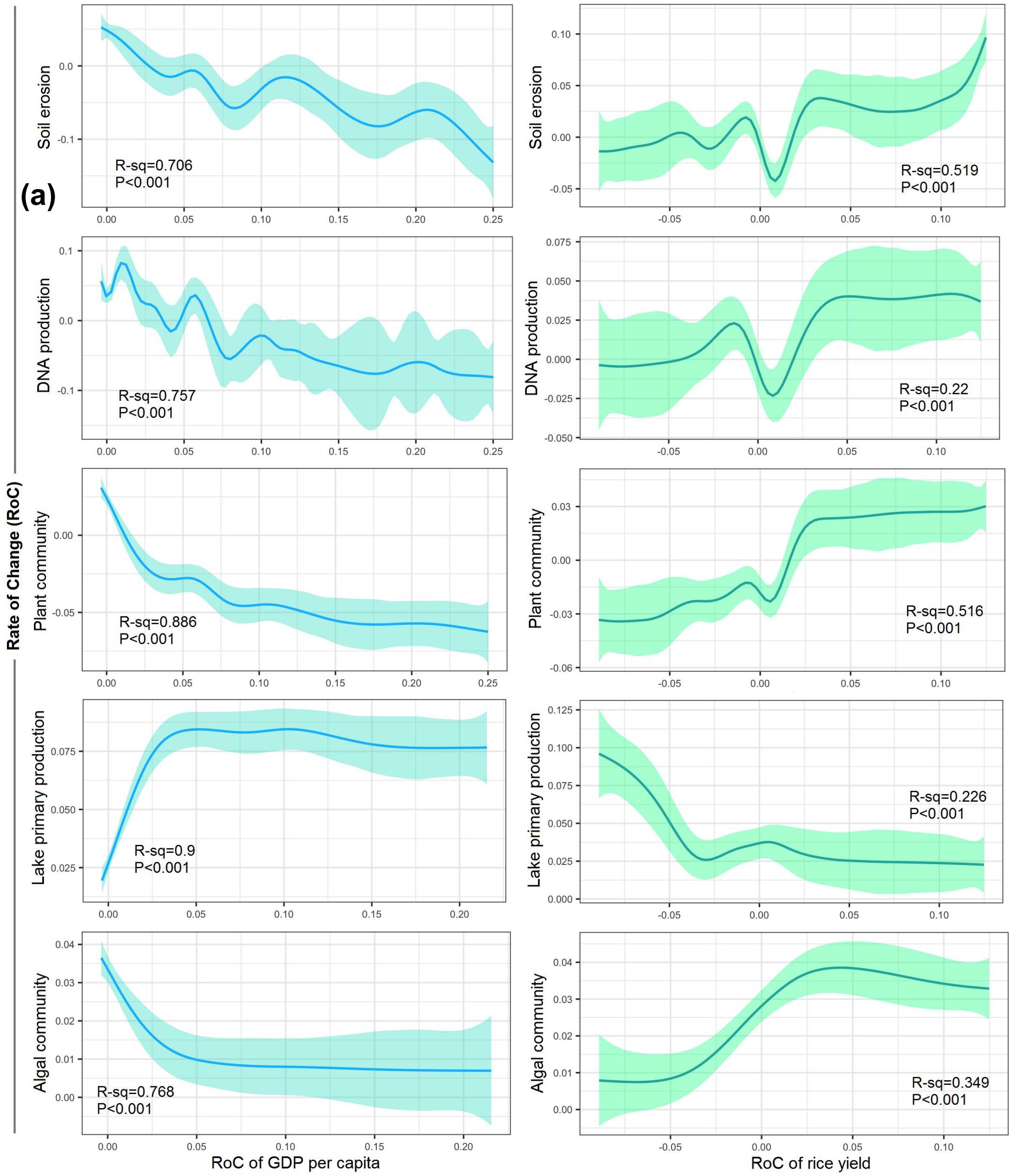

Fig. S13. The fitted smooth functions from GAMs analysis for the relationships between socioeconomic and ecological RoCs. The additive model fitted to five ecological variable, with the probability levels obtained for two key socioeconomic drivers is presented. The considered socioeconomic variables are RoCs in industry-dominated economy (GDP per capita) and agriculture production (rice yield) from the study region. The considered ecological response variables are RoCs in soil erosion (magnetic susceptibility), DNA production (*sed*aDNA concentration), terrestrial plant community (NMDS score of plant *sed*aDNA), lake primary production (chlorophylls), and algal community (NMDS score of pigments) inferred from the lake sediment paleorecords. Shaded areas are 95% confidence intervals on the estimated effect. Statistical summary for the GAMs is shown in *SI Appendix*, Table S2.

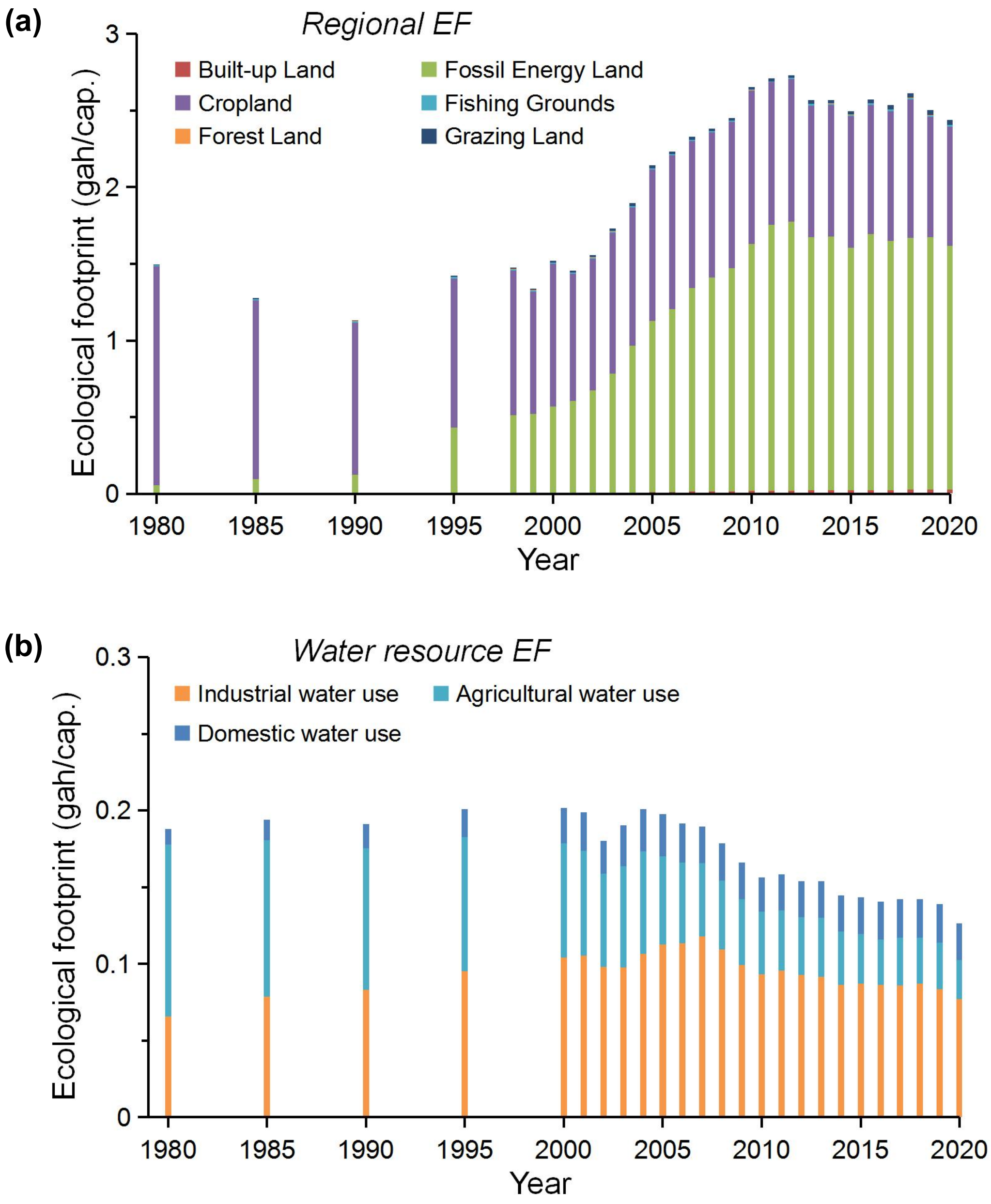

Fig. S14. Ecological footprint per capita and its components during 1980–2020 in the study region. Changes in ecological footprint related to human land-use in Jiangsu Province (a) and freshwater resource consumption in Lake Taihu watershed (b) over time have been shown.

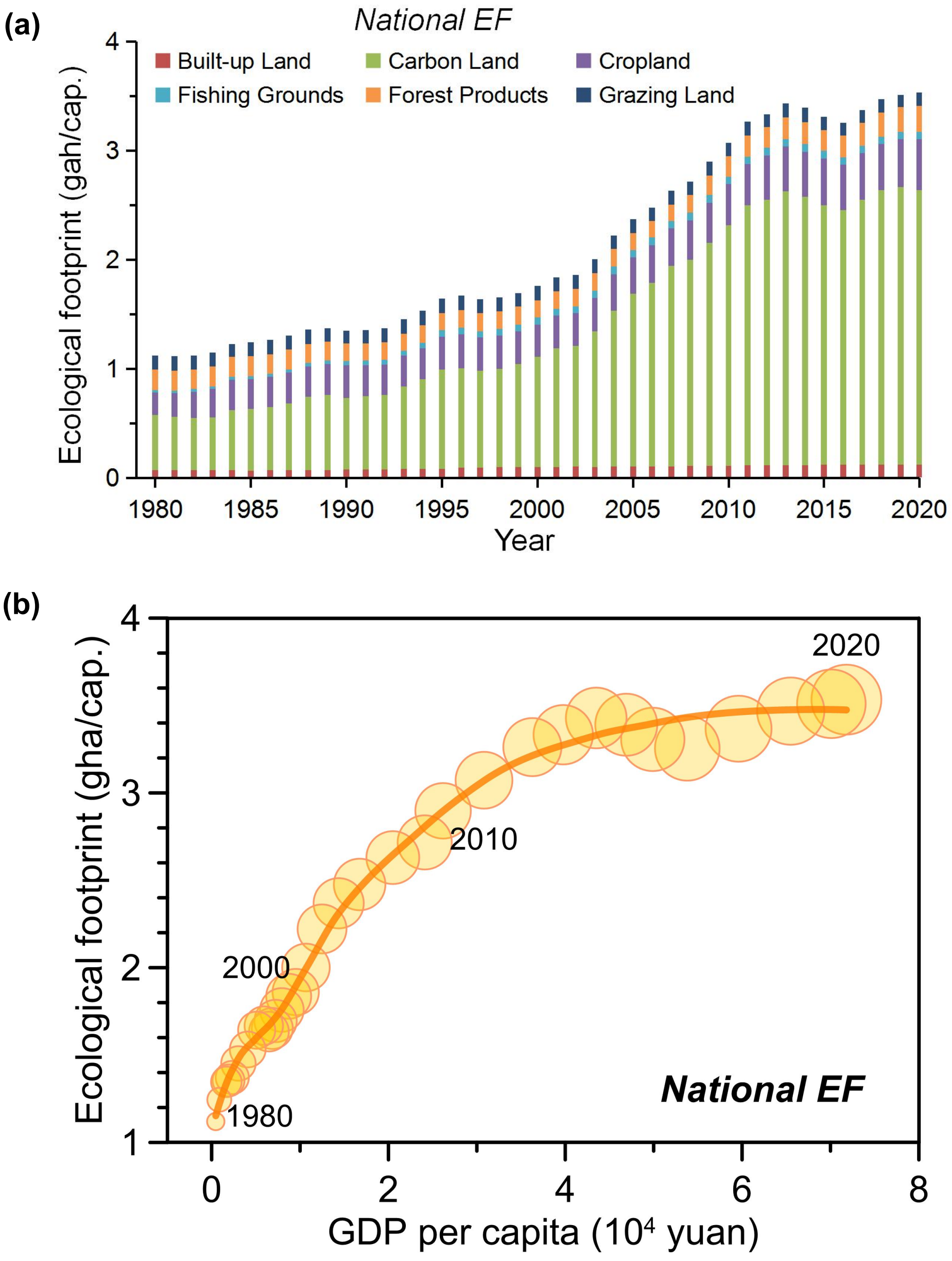

Fig. S15. National ecological footprint (EF) per capita of China and its relationships with economic growth during 1980–2020. (a) Changes in national EF and its components over time (37). (b) Relationships between national ecological footprint (EF) per capita and GDP per capita of China with LOESS fit. The size of each data point represents the corresponding date (year), which is labeled around the data point. This graph generally shows synchronous increases in per capita GDP and ecological footprint at the national level, which shows limited decoupling trends of ecological footprint from economic growth in recent years.

Table S1. Statistical summary of GAM-based trends fitted to key socioeconomic, biophysical and climate variables of Lake Taihu SES through time. This table is a supplement of *SI Appendix*, Figs. S11 and S12. Biophysical dynamics (ecological degradation) regarding to anthropogenic soil erosion, primary production, and community turnover, are indicated by paleoecological time series of sediment mass magnetic susceptibility, total *seda*DNA concentration, chlorophylls concentration, and NMDS scores of terrestrial plant community (*seda*DNA) and aquatic algal community (pigments), respectively. The time-series of these variables are z-score normalized before model analysis. Model performance is indicated by the R-sq.(adj) and deviance explained (dev. expl.) of the respective models.

Process	Variable	edf	F	P	R-sq.(adj)	Dev. expl.
Socioeconomic development	Population	13.61	10006	<2e-16***	1	100%
	Rice yield	13.92	31.79	<2e-16***	0.900	92.4%
	GDP per capita	21.03	5078	<2e-16***	1	100%
	Industrial output	13.73	3063	<2e-16***	0.999	99.9%
Biophysical dynamics (ecological degradation)	Soil erosion	11.26	70.64	<2e-16***	0.915	92.6%
	DNA production	4.241	12.83	<2e-16***	0.725	77.8%
	Plant community	3.01	0.98	0.325	0.151	26.7%
	Lake production	4.689	67.15	<2e-16***	0.909	92.0%
	Algal community	3.863	68.22	<2e-16***	0.884	89.5%
Climate change	Temperature	7.212	40.47	<2e-16***	0.752	76.7%
	Precipitation	7.897	2.371	0.0144*	0.158	21.4%

For all variables in the models, the estimated degrees of freedom (edf) and F values with corresponding significance levels (***p<0.001, **p<0.01, *p<0.05,) are shown.

Table S2. GAM analysis summary for the relationships between socioeconomic and ecological RoCs in *SI Appendix*, Fig. S13. The summary for the additive model fitted to the ecological variable, with the probability levels obtained for two major socioeconomic drivers is presented. The considered socioeconomic variables are RoCs in industry-dominated economy (GDP per capita) and agriculture production (rice yield) from the watershed. The considered ecological response variables are RoCs in soil erosion (magnetic susceptibility), DNA production (*sed*aDNA concentration), terrestrial plant community (NMDS score of plant DNA), lake production (chlorophylls) and algal community (NMDS score of pigments) inferred from the lake sediment paleorecords. Model performance is indicated by the R-sq.(adj) and deviance explained (dev. expl.) of the respective models..

**Socioeconomic variable**	**Ecological response**	**edf**	**F**	**P**	**R-sq.(adj)**	**Dev. expl.**
GDP per capita	Soil erosion	13.000	13.580	<2e-16***	0.706	76.2%
	DNA production	20.080	9.707	<2e-16***	0.757	82.8%
	Plant community	13.000	41.640	<2e-16***	0.886	90.8%
	Lake production	25.000	24.860	<2e-16***	0.9	93.8%
	Algal community	25.000	9.762	<2e-16***	0.768	85.6%
Rice yield	Soil erosion	10.290	8.308	5.48e-12***	0.519	56.9%
	DNA production	7.072	3.227	0.00187***	0.22	27.6%
	Plant community	4.276	20.440	9.32e-16***	0.516	53.8%
	Lake production	4.285	5.648	0.000118***	0.226	26.0%
	Algal community	4.637	9.440	6.28e-08***	0.349	38.1%

For all variables in the models, the estimated degrees of freedom (edf) and F values with corresponding significance levels (***p<0.001) are shown.

Table S3. Paleoenvironmental indicator records used for determining the dominant eco-environmental variability of Taihu lake-watershed system.

Subsystem	Ecological process or state	Sediment proxy record	Known driver
Watershed land system	Soil erosion	Mass magnetic susceptibility	Land-use change; cultivated slope; flood
	DNA/biomass production	Total sedDNA concentration	Fertilizers use; cultivation; erosion; climate change
	Plant community turnover	NMDS score of plant sedDNA	Agricultural practice; urbanization; policy
Lake ecosystem	Lake primary production/ productivity	Chlorophylls concentration	Fertilizer use; erosion; raw sewage input; warming
	Algal community turnover	NMDS score of pigment assemblages	Nutrient enrichment; climate change; hydrological fluctuations

Table S4. Ecological footprint (EF) measurement indicators and data description during 1980–2020.

**Ecological footprint account**	**Consumption indicator**	**Land-use cover type**	**Data source**
Biological resources	Cereal	Cropland	Statistical Yearbooks of Jiangsu Province<br>National Bureau of Statistics of China<br>Food and Agriculture Organization of the United Nations (FAO)
	Vegetables	Cropland	
	Pork	Cropland	
	Poultry	Cropland	
	Eggs	Cropland	
	Beef	Grazing land	
	Mutton	Grazing land	
	Milk	Grazing land	
	Wood	Forest land	
	Bamboo	Forest land	
	Fruit	Forest land	
	Aquatic products	Inland fishing grounds	
Fossil energy resources	Electricity	Built-up land	
	Raw coal	Fossil energy land	
	Hard coke	Fossil energy land	
	Crude oil	Fossil energy land	
	Gasoline	Fossil energy land	
	Coal oil	Fossil energy land	
	Diesel oil	Fossil energy land	
	Fuel oil	Fossil energy land	
	Liquefied petroleum gas	Fossil energy land	
Water resources	Freshwater resources (Industrial, agricultural, and domestic water use)	Water areas (lakes, rivers, reservoir etc.)	Taihu Basin Authority, China

Note: (i) Data of the global average production capacity of above biological resources (kg ha^{-1}) is sourced from the United Nations (UN) Food and Agriculture Organization (https://www.fao.org/faostat/en/#data/QCL). (ii) Data of the global average production capacity of freshwater resources (m^3 ha^{-1}) is sourced from the "State of Global Water Resources Report 2022" of the World Meteorological Organization (https://public.wmo.int/our-mandate/waters/state-of-global-water-resources-2022). (iii) Equivalence factor time series for biological resource and water resource accounts are sourced from the Global Footprint Network (37).

Table S5. Parameters for fossil energy footprint account.

Item	Conversion Factor (GJ t^{-1})	Global Average EF(GJ ha^{-1})
Raw coal	20.908	55
Hard coke	28.435	55
Crude oil	41.816	93
Gasoline	43.07	93
Coal oil	43.07	93
Diesel oil	42.552	93
Fuel oil	41.816	93
Liquefied petroleum gas	50.179	71
Electricity	0.0036	1000

Note: (i) The electricity consumption was converted into built-up land area, the other energy consumption types were converted into fossil production land area, and all the consumed energy was converted into fossil land area according to the heat conversion coefficient used in global energy statistics (38) and the FOA statistical databases. (ii) The unit of the conversion coefficient of electricity is GJ $(kW\cdot h)^{-1}$.

SI References

1. E. C. Ellis, S. Wang, Sustainable traditional agriculture in the Tai Lake region of China. *Agric. Ecosyst. Environ.* **61,** 177–193 (1997).
2. X. Xu *et al.*, Impacts of land use changes on net ecosystem production in the Taihu Lake Basin of China from 1985 to 2010. *J. Geophys. Res. Biogeosci.* **122,** 690–707 (2017).
3. N. L. Rose *et al.*, Sedimentary evidence for changes in the pollution status of Taihu in the Jiangsu region of eastern China. *J. Paleolimnol.* **32,** 41–51 (2004).
4. Q. Lin *et al.*, Deciphering centurial anthropogenic pollution processes in large lakes dominated by socio-economic impacts. *Anthropocene* **32,** 100269 (2020).
5. Y. Ge, K. Zhang, X. Yang, A 110-year pollen record of land use and land cover changes in an anthropogenic watershed landscape, eastern China: Understanding past human-environment interactions. *Sci. Total Environ.* **650,** 2906–2918 (2019).
6. S. McGowan, Pigments studies, p. 326–338. In S. Elias [ed.], The encyclopedia of quaternary science, v. 3, 2nd ed. Elsevier (2013).
7. N. Chen, T. S. Bianchi, B. A. McKee, J. M. Bland, Historical trends of hypoxia on the Louisiana shelf: Application of pigments as biomarkers. *Org. Geochem.* **32,** 543–561 (2001).
8. P. R. Leavitt, A review of factors that regulate carotenoid and chlorophyll deposition and fossil pigment abundance. *J. Paleolimnol.* **9,** 109–127 (1993).
9. P. R. Leavitt, R. D. Vinebrooke, D. B. Donald, J. P. Smol, D. W. Schindler, Past ultraviolet radiation environments in lakes derived from fossil pigments. *Nature* **388,** 457–459 (1997).
10. M. A. Stevenson *et al.*, Impacts of forestry planting on primary production in upland lakes from north-west Ireland. *Glob. Chang. Biol.* **22,** 1490–1504 (2016).
11. W. Chen, G. F. Ficetola, Numerical methods for sedimentary-ancient-DNA-based study on past biodiversity and ecosystem functioning. *Environ. DNA* **00,** 1–15 (2020).
12. L. Zhang, Z. Luo, D. Mallon, C. Li, Z. Jiang, Biodiversity conservation status in China's growing protected areas. *Biol. Conserv.* **210,** 89–100 (2017).
13. Y. Li, L. Zhou, H. Cui, Pollen indicators of human activity. *Chin. Sci. Bull.* **53,** 991–1002 (2008).
14. S. Qu, L. Wang, A. Lin, H. Zhu, M. Yuan, What drives the vegetation restoration in Yangtze River basin, China: climate change or anthropogenic factors? *Ecol. Indic.* **90,** 438–450 (2018).
15. J. A. Dearing *et al.*, Social-ecological systems in the Anthropocene: the need for integrating social and biophysical records at regional scales. *Anthropol. Rev.* **2,** 1–27 (2015).
16. J. P. Smol, Under the radar: long-term perspectives on ecological changes in lakes. *Proc. Biol. Sci.* **286,** 20190834 (2019).
17. E. Capo *et al.*, Lake sedimentary DNA research on past terrestrial and aquatic biodiversity: Overview and recommendations. *Quaternary* **4,** 6 (2021).
18. X. Boës, J. Rydberg, A. Martinez-Cortizas, R. Bindler, I. Renberg, Evaluation of conservative lithogenic elements (Ti, Zr, Al, and Rb) to study anthropogenic element enrichments in lake sediments. *J. Paleolimnol.* **46,** 75–87 (2011).
19. M. Kylander, L. Ampel, B. Wohlfarth, D. Veres, High-resolution X-ray fluorescence core scanning analysis of Les Echets (France) sedimentary sequence: new insights from chemical proxies. *J. Quat. Sci.* **26,** 109–117 (2011).

20. F. Arnaud *et al.*, Erosion under climate and human pressures: an alpine lake sediment perspective. *Quatern. Sci. Rev.* **152,** 1–18 (2016).

21. F. Oldfield, Environmental magnetism – a personal perspective. *Quat. Sci. Rev.* **10,** 73–85 (1991).

22. K. Yu et al., Climate change and soil erosion in a small alpine lake basin on the Loess Plateau, China. *Earth Surf. Process. Landforms* **42,** 1238–1247.

23. I. Domaizon, A. Winegardner, E. Capo, J. Gauthier, I. Gregory-Eaves, DNA-based methods in paleolimnology: new opportunities for investigating long-term dynamics of lacustrine biodiversity. *J. Paleolimnol.* **58,**1–21 (2017).

24. I. G. Alsos *et al.*, Plant DNA metabarcoding of lake sediments: How does it represent the contemporary vegetation. *PLoS ONE* **13,** e0195403 (2018).

25. C. Giguet-Covex *et al.*, New insights on lake sediment DNA from the catchment: importance of taphonomic and analytical issues on the record quality. *Sci. Rep.* **9,** 14676 (2019).

26. G. Pietramellara *et al.*, Extracellular DNA in soil and sediment: fate and ecological relevance. *Biol. Fertil. Soils* **45,** 219–235 (2009).

27. L. Parducci *et al.*, Ancient plant DNA in lake sediments. *New Phytol.* **214,** 924–942 (2017).

28. S. R. Carpenter *et al.*, Early warnings of regime shifts: a whole-ecosystem experiment. *Science* **332,** 1079–1082 (2011).

29. H. Maheux, P. R. Leavitt, L. J. Jackson, Asynchronous onset of eutrophication among shallow prairie lakes of the Northern Great Plains, Alberta, Canada. *Glob. Chang. Biol.* **22,** 271–283 (2016).

30. H. L. Moorhouse *et al.*, Regional versus local drivers of water quality in the Windermere catchment, Lake District, United Kingdom: The dominant influence of wastewater pollution over the past 200 years. *Glob. Chang. Biol.* **24,** 4009–4022 (2018).

31. Q. Lin, K. Zhang, S. McGowan, E. Capo, J. Shen, Synergistic impacts of nutrient enrichment and climate change on long-term water quality and ecological dynamics in contrasting shallow-lake zones. *Limnol. Oceanogr.* **66,** 3271–3286 (2021).

32. H. A. Ewing *et al.*, “New” cyanobacterial blooms are not new: Two centuries of lake production are related to ice cover and land use. *Ecosphere* **11,** e03170 (2020).

33. P. Taberlet, E. Coissac, F. Pompanon, C. Brochmann, E. Willerslev, Towards next-generation biodiversity assessment using DNA metabarcoding. *Mol. Ecol.* **21,** 2045–2050 (2012).

34. J. A. Dearing *et al.*, Extending the timescale and range of ecosystem services through paleoenvironmental analyses: the example of the lower Yangtze basin. *Proc. Natl. Acad. Sci. U. S. A.* **109,** E1111–E1120 (2012).

35. M. Cuenca-Cambronero *et al.*, An integrative paleolimnological approach for studying evolutionary processes. *Trends Ecol. Evol.* **37,** 488–496 (2022).

36. E. C. Grimm, Tilia 1.7.16 Software. Springfield, Illinois State Museum, Research and Collection Center (2011).

37. York University Ecological Footprint Initiative & Global Footprint Network. Public Data Package of the National Footprint and Biocapacity Accounts, 2023 edition. Produced for the Footprint Data Foundation and distributed by Global Footprint Network. Available online at: https://data.footprintnetwork.org.

38. D. Qiu, Energy Planning & Systematic Analysis. Tsinghua University Press: Beijing, China (1995).
